# De novo design of modular protein hydrogels with programmable intra- and extracellular viscoelasticity

**DOI:** 10.1073/pnas.2309457121

**Published:** 2024-01-30

**Authors:** Rubul Mout, Ross C. Bretherton, Justin Decarreau, Sangmin Lee, Nicole Gregorio, Natasha I. Edman, Maggie Ahlrichs, Yang Hsia, Danny D. Sahtoe, George Ueda, Alee Sharma, Rebecca Schulman, Cole A. DeForest, David Baker

**Affiliations:** ^a^Department of Biochemistry, University of Washington, Seattle, WA 98195; ^b^Institute for Protein Design, University of Washington, Seattle, WA 98195; ^c^Stem Cell Program at Boston Children’s Hospital, Harvard Medical School, Boston, MA 02115; ^d^Department of Bioengineering, University of Washington, Seattle, WA 98195; ^e^Department of Chemical Engineering, University of Washington, Seattle, WA 98195; ^f^Institute for Stem Cell and Regenerative Medicine, University of Washington, Seattle, WA 98195; ^g^Department of Chemistry, University of Washington, Seattle, WA 98195; ^h^Molecular Engineering & Sciences Institute, University of Washington, Seattle, WA 98195; ^i^Molecular and Cellular Biology Graduate Program, University of Washington, Seattle, WA 98195; ^j^Medical Scientist Training Program, University of Washington, Seattle, WA 98195; ^k^HHMI, University of Washington, Seattle, WA 98195; ^l^College of Professional Studies, Northeastern University, Boston, MA 02115; ^m^Department of Chemical and Biomolecular Engineering, Johns Hopkins University, Baltimore, MD 21218; ^n^Department of Computer Science, Johns Hopkins University, Baltimore, MD 21218

**Keywords:** protein hydrogels, de novo protein design, viscoelasticity, biomolecular condensate, self-assembly

## Abstract

Relating the macroscopic properties of protein-based materials to their underlying component microstructure is an outstanding challenge. Here, we exploit computational design to specify the size, flexibility, and valency of de novo protein building blocks, as well as the interaction dynamics between them, to investigate how molecular parameters govern the macroscopic viscoelasticity of the resultant protein hydrogels. We construct gel systems from pairs of symmetric protein homo-oligomers, each comprising 2, 5, 24, or 120 individual protein components, that are crosslinked either physically or covalently into idealized step-growth biopolymer networks. Through rheological assessment, we find that the covalent linkage of multifunctional precursors yields hydrogels whose viscoelasticity depends on the crosslink length between the constituent building blocks. In contrast, reversibly crosslinking the homo-oligomeric components with a computationally designed heterodimer results in viscoelastic biomaterials exhibiting fluid-like properties under rest and low shear, but solid-like behavior at higher frequencies. Exploiting the unique genetic encodability of these materials, we demonstrate the assembly of protein networks within living mammalian cells and show via fluorescence recovery after photobleaching (FRAP) that mechanical properties can be tuned intracellularly in a manner similar to formulations formed extracellularly. We anticipate that the ability to modularly construct and systematically program the viscoelastic properties of designer protein-based materials could have broad utility in biomedicine, with applications in tissue engineering, therapeutic delivery, and synthetic biology.

## Significance

Protein-based hydrogels have many applications in cellular engineering and medicine. Most genetically encodable protein hydrogels are made from naturally occurring proteins or protein–polymer hybrid constructs. Here, we describe de novo protein hydrogels and systematically investigate the impact of microscopic properties of the building blocks (e.g., supramolecular interaction, valencies, geometries, flexibility) on the resultant macroscopic gel mechanics, both intra- and extracellularly. These de novo supramolecular protein assemblies, whose properties vary from viscoelastic fluids to gels, provide expanded opportunities for applications in synthetic biology and medicine.

Hydrogels are water-swollen (bio)polymer networks that are widely exploited for bioengineering and medical applications including tissue engineering and drug delivery (1–6). The vast majority of hydrogels explored to date have been created from synthetic polymers [e.g., poly(ethylene glycol), poly(hydroxyethylmethacrylate)] (7–10), or naturally derived proteins (e.g., collagen, fibrin) (11–13). Though such materials have profound utility across many fields, the precursor polydispersity inherent to these systems and a lack of well-defined secondary structure introduces concerns over batch-to-batch variability and imposes challenges in specifying gel attributes a priori. Sidestepping many of these limitations, monodisperse recombinant proteins of various kinds have been exploited to create hydrogels through covalent and noncovalent interactions (14–21). While these systems have afforded some degree of control over network mechanics (22), prior approaches utilizing native (primarily unstructured) proteins have not permitted systematic investigation of how molecular characteristics of the building blocks (e.g., supramolecular interaction, valencies, geometries, and flexibility) influence macroscopic material properties.

De novo protein design provides an attractive avenue to computationally specify and vary the molecular characteristics of material building blocks (23). Guided by the physical principles that underlie protein folding, tools such as Rosetta (24) have made it possible to design functional proteins with user-specified secondary, tertiary, and quaternary structures. Rosetta has been used to create self-assembling symmetric protein nanomaterials in which the atomic and microscopic properties of the constituents dictate macromolecular structure. These include bounded systems with cyclic (25), dihedral (26), tetrahedral (27), octahedral (28), and icosahedral symmetry consisting of 60 or 120 subunits (29, 30), as well as structurally unbounded 1D fibers (31), 2D arrays (32), and 3D peptide crystals (33), with 3D structures confirmed by X-ray crystallography and cryoelectron microscopy. Though such atomically precise structures have found utility across a wide variety of applications including the rational design of synthetic vaccines (34, 35), modulating cell signaling (36), and molecular motors (37), these methods have not yet been applied towards the creation of bulk materials including hydrogels.

Hypothesizing that de novo designed protein nanostructures could be utilized as branched material crosslinkers with user-specified multivalency, we sought to create biopolymer gels from these computationally designed protein components and to utilize these precisely defined materials to probe the effect of individual microscopic precursor parameters on macroscopic material properties (e.g., viscoelasticity). We anticipated that by exploiting the genetic encodability of this recombinant protein-based approach, such hydrogel precursors could be modularly defined in a plug-and-play manner through conventional cloning alterations of the expression vectors, enabling facile modulation of the length, rigidity, density, type, and overall valency of the material crosslinkers. Moreover, since the crosslinking species themselves can be designed to have precise structure and symmetry, we anticipated that this strategy would yield more idealized and molecularly homogenous materials than those offered by conventional hydrogel fabrication.

## Results

### Design of De Novo Protein Hydrogels with Programmable Viscoelasticity.

**Fig. 1.**
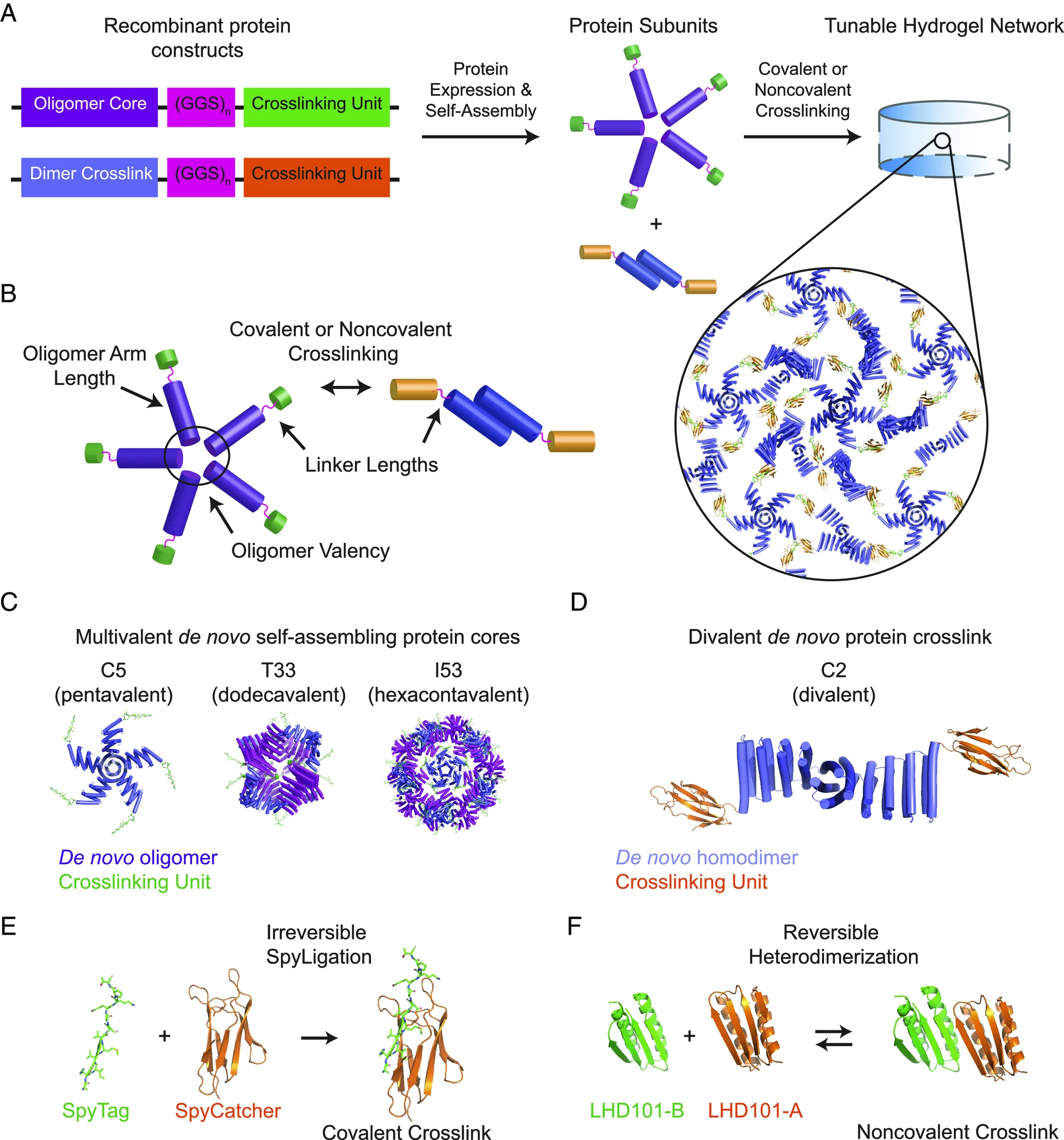
Multi-scale design of de novo protein hydrogel networks. (*A*) Protein fusions consisting of self-assembling oligomeric cores fused to crosslinking units are linked by a flexible (GGS)_n_ linker (*Left*) and expressed recombinantly to produce oligomer cores and dimer crosslinks (*Center*), which can then be mixed to form a tunable hydrogel network (*Right*). (*B*) Mechanisms for network tunability. PDB models depicting three multimeric de novo protein cores, showing pendant crosslinking units (here SpyTag) (*C*); the end-functionalized dimeric protein crosslink (shown here with SpyCatcher) (*D*); SpyCatcher/SpyTag covalent crosslinking chemistry (*E*); and noncovalent crosslinking by reversible heterodimerization of the de novo LHD101A/B pair (*F*).

We envisioned creating well-defined protein hydrogels through step-growth polymerization methodologies, whereby symmetric homo-oligomers serving as multifunctional network branch points with defined valency would be crosslinked physically or chemically with a reactive protein homodimer (Fig. 1). Multivalent building blocks were selected from a de novo designed homopentamer (C5, 118.8 kDa as assembled, diameter = 11.3 nm) (26), a 24-chain tetrahedral nanocage (T33, 529 kDa as assembled, diameter = 15.5 nm) (38), and a 120-chain icosahedral nanocage (I53, 1,867.3 kDa as assembled, diameter = 24 nm) (38). To form bulk gels from these oligomeric building blocks, we employed a designed homodimer as a divalent material crosslinker with two symmetrical termini pointing in opposite directions (C2, 59 kDa as assembled) (26). C2 and C5 have helical bundles at the core and exterior protruding ‘arms’ that can be readily extended while preserving structural rigidity by the incorporation of additional designed repeat modules (26). Covalent crosslinking of hydrogel precursors (i.e., C2 with either C5, T33, or I53) was conducted via SpyLigation, a protein “superglue” chemistry, in which an isopeptide bond is irreversibly formed between evolved SpyCatcher- (SC, 88 amino acid residue, 9.5 kDa) and SpyTag- (ST, 13 amino acids) modified species (39). Alternatively, hydrogel precursors were crosslinked through the non-covalent association of designed protein heterodimers LHD101-A and LHD101-B (respectively, denoted as HA and HB for heterodimers A and B) (40).

To create bifunctional de novo protein crosslinkers, we first genetically fused each of the self-assembling C2 subunits to SC through a variable-length flexible linker with sequence (Gly-Gly-Ser)_n_ [(GGS)_n_ with n = 1 or 5]. C5, T33, and I53 were similarly fused to ST through a (GGS)_n_ linker (n = 1, 5, or 10), yielding multivalent self-assembled protein cores that displayed 5, 12, or 60 ST handles, respectively. For the assemblies crosslinked through the non-covalent association of the designed protein heterodimers, only the C2-(GGS)_5_-HA: C5-(GGS)_5_-HB pair was chosen. For clarity, we employ a standard naming convention: Protein assemblies are denoted “X-Y-Z”, where “X” is the underlying multivalent protein core, “Y” is the number of GGS repeats in the flexible linker, and “Z” is the crosslinking functional group (e.g., I53-5-ST refers to the I53 core with 5 GGS repeats between it and each of the 60 total ST functional groups). Following plasmid construction through standard cloning techniques, 6xHis-tagged proteins were expressed recombinantly in Lemo *Escherichia coli*, solubly purified by Ni-NTA immobilized-metal affinity chromatography, buffer exchanged into tris-buffered saline (300 mM NaCl, 25 mM Tris, pH 8.0), and concentrated as self-assembled complexes to 100 to 300 mg/mL (26). Protein purity was confirmed by Polyacrylamide gel electrophoresis (SDS-PAGE) analysis.

Bulk hydrogels were formed by mixing multimeric protein components to a fixed final concentration (10% w/v) with equal stoichiometry between reactive end groups (i.e., SC and ST, HA and HB). Solution viscosity noticeably increased within seconds of component mixing, consistent with rapid SpyLigation kinetics (39) and large heterodimer binding affinities (40). To ensure complete gelation, network formation was allowed to proceed at room temperature (25 °C) for >6 h, and cross-linking was analyzed by SDS-PAGE (*SI Appendix*, Fig. S1) prior to bulk materials characterization. Free-standing macroscopic gels were obtained for all tested combinations.

#### Varying flexible linker length.

**Fig. 2.**
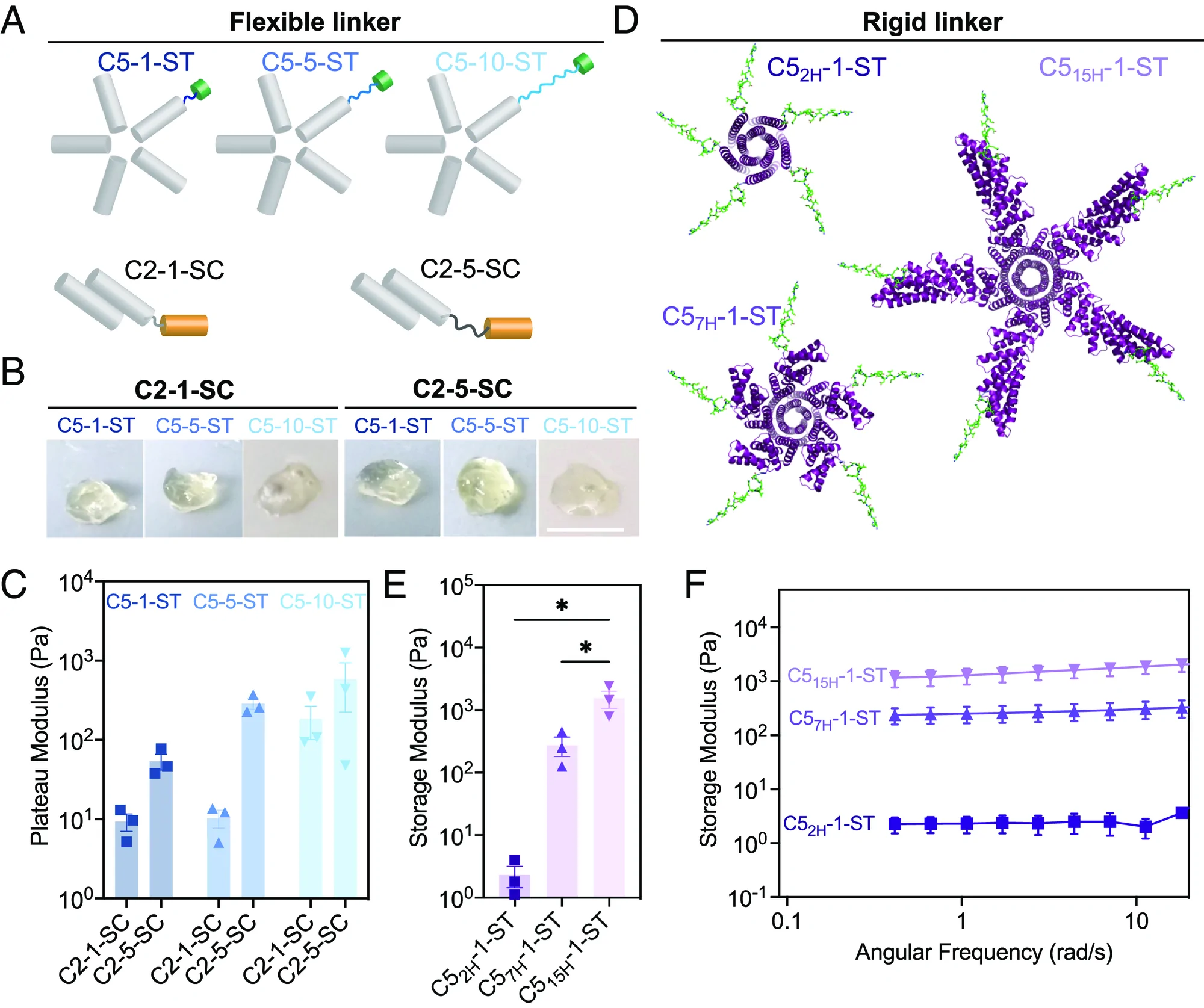
Influence of linker length and rigidity on covalent hydrogel viscoelasticity. The mechanical properties of the hydrogels are dictated by the lengths of the linker connecting the interacting domains. (*A*−*C*) Flexible linkers. (*A*) Schematics showing C5 and C2 helical bundle oligomers with variable (GGS)_n_ linker. (*B*) Image of hydrogels formed by C2-1-SC and C2-5-SC with ST (SpyTagged) C5 (i.e., C5-1-ST; C5-5-ST; and C5-10-ST]. (Scale bar, 4 mm.) (*C*) Storage modulus of various C2 × C5 combinations. (*D*–*F*) Rigid connecting elements. (*D*) PDB models of C5 oligomers with 2, 7, and 15 helical lengths. (*E*) Storage modulus of C2-1-SC × C5_nH_-1-ST (rigid arm) combinations derived from angular frequency sweep (*F*). Error bars indicate ± SEM. *P < 0.05 by one-way ANOVA and Holm-Sidak post hoc test.

To assess how the flexibility of the linkers connecting the components affect material viscoelasticity, we measured the rheological properties of gels made from different combinations of C2 × C5 with different length flexible linkers between the rigid homo-oligomers and the covalent SC/ST crosslink (Fig. 2*A*). Representative images of hydrogels formed 12 h after mixing the components are shown in Fig. 2*B*. Then, 0.5-mm-thick hydrogel droplets with an 8 mm diameter were subjected to frequency sweeps between parallel plates ranging from 0.1 to 500 rad/s at a constant maximum strain of 10%, which was empirically determined to lie within the linear viscoelastic regime of these hydrogels. The stiffness of the hydrogel (measured through storage modulus within the linear viscoelastic region) increased as the length of the (GGS)_n_ linker increased (Fig. 2*C*).

#### Varying rigid arm length.

We next investigated how the size of structured hydrogel components affect the rheological properties of the gels. We took advantage of the ability to computationally vary the length of the rigid helical repeat arms extending from the helical bundle central cores. Each repeat, comprised of two helices (41), is encoded by an identical sequence of 42 amino acids, and the length of the rigid arms can be varied modularly simply by inserting or deleting sequence repeats. Keeping the flexible interaction domain (SC/ST) at a minimum distance of (GGS)_1_, we generated a series of pentameric protein complexes with arm lengths of 2 helices (C5_2H_-1-ST, only the core pentameric helical bundle without extra repeat extension), 7 helices (C5_7H_-1-ST), and 15 helices (C5_15H_-1-ST), respectively (Fig. 2*D*), and again measured gel mechanics through frequency sweep rheology. The rigid arm length from the core varies depending on the repeat extension: ~1 nm for C5_2H_; ~3.7 nm for C5_7H_; and ~7.5 nm for C5_15H_. Similar to the results with varying flexible linker length, we observed a steep increase in the storage modulus as the rigid arm length increased from 1.0 to 7.5 nm (Fig. 2*E*). In addition, networks formed by all the rigid arm constructs maintained viscoelastic properties over a range of frequencies (Fig. 2*F*).

#### Varying valency.

**Fig. 3.**
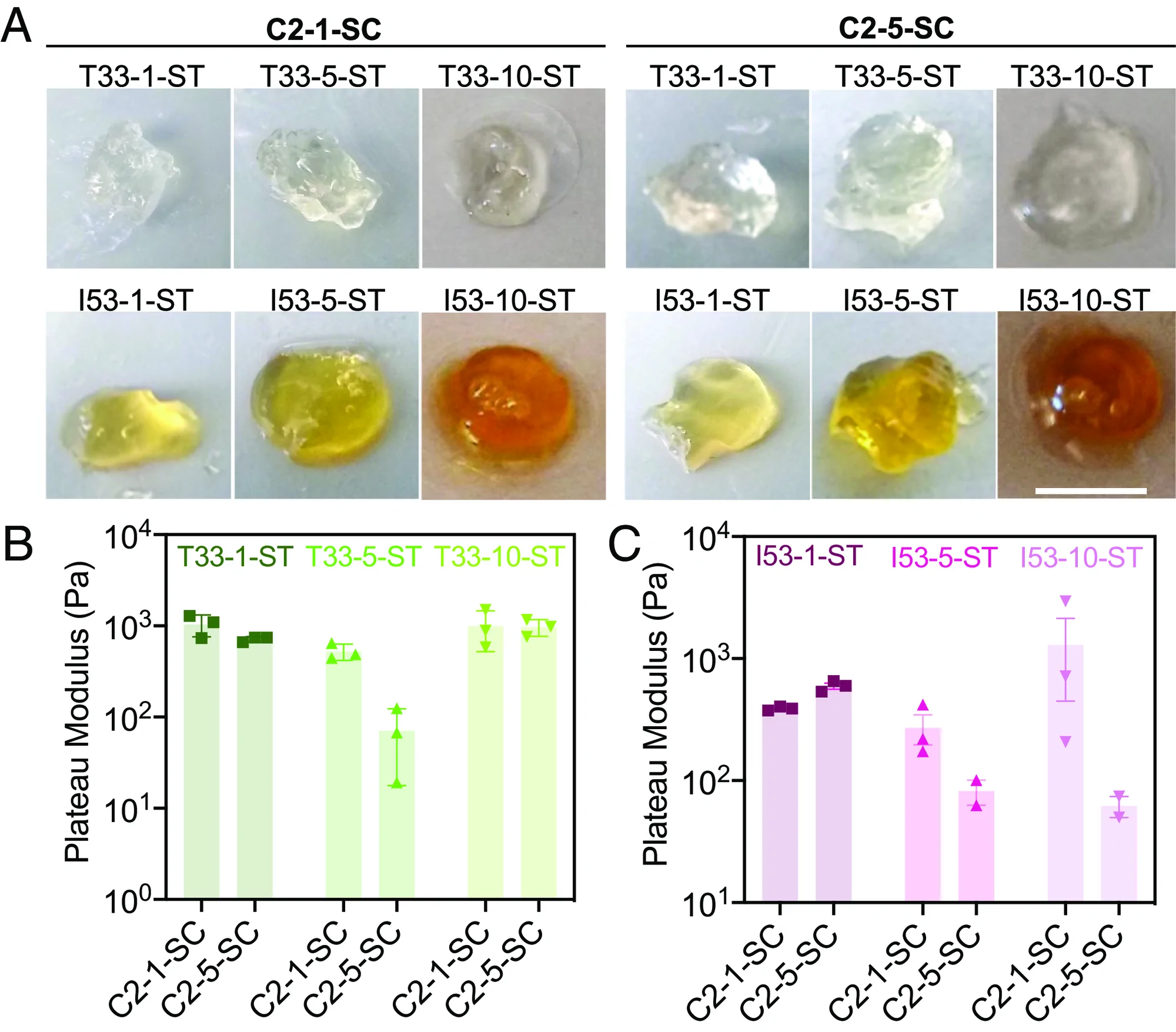
Effect of component valency on hydrogel properties. The elastic modulus of the hydrogels made from higher valency protein assemblies generally decreased as the length of the linker connecting the interacting domains of C2 increased, unlike in the C5 case (Fig. 2*C*). (*A*) Gels formed by C2 × T33 and C2 × I53 combinations. (Scale bar, 4 mm.) (*B* and *C*) Storage moduli of combinations of C2 × T33 (*B*) or C2 × I53 (*C*) with different flexible linker lengths. Note that slight variations in gel colors were observed, attributed to differences in protein tryptophan contents ((42)).

We next investigated how building block valency affected hydrogel properties. We replaced the C5 SpyCatcher tagged component with one of two two-component protein nanocages—a tetrahedral (T33 symmetry, 24 chains, 12 ST) and an icosahedral (I53 symmetry, with 120 chains and 60 ST). To assemble hydrogels, these nanocage-STs were mixed with C2-SC with different (GGS)_n_ linker lengths. Gel formation was observed for all combinations after mixing, but with starkly different kinetics. The C2 × I53 combinations exhibited rapid gel formation in minutes, while the C2 × T33 combinations formed gels on the hour time scale (Fig. 3*A*; 40 microliters of each combination after 12 h were imaged using a regular camera). Rheological measurement of the elastic moduli revealed that, for both C2 × T33 and C2 × I53 combinations, the storage modulus dropped as the (GGS)_n_ linker length increased (Fig. 3 *B* and *C*), in contrast to the results with the smaller C5 core, where stiffness increased as the linker length increased (Fig. 2*C*). In networks formed with higher-valency structured components, gel rigidity may no longer be limited by the extent of the SC/ST reaction; instead, increasing linker length likely reduces network stiffness by giving more flexibility to regions between the large oligomeric protein cores.

**Fig. 4.**
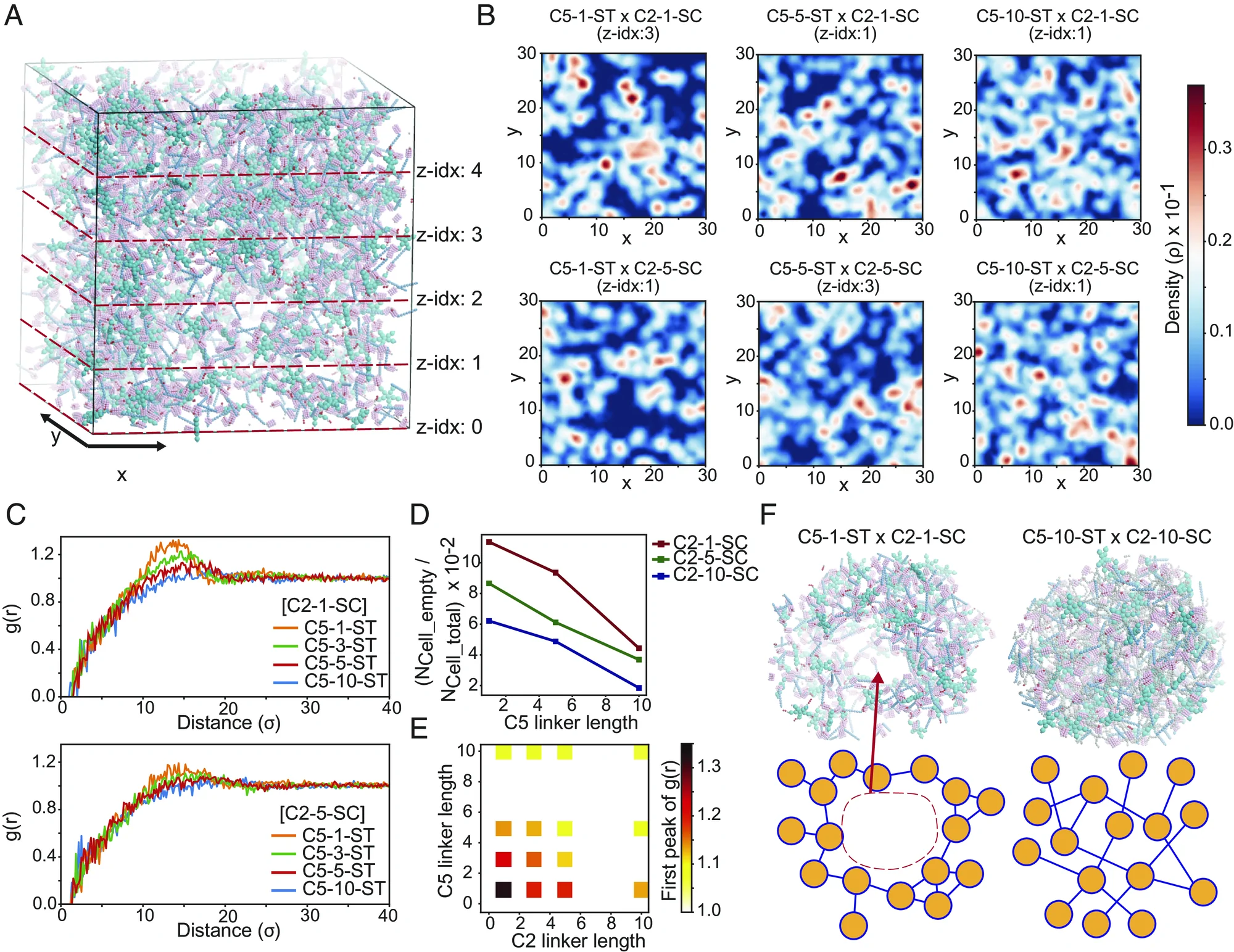
MD simulation of covalently cross-linked de novo protein hydrogels. (*A*) A system of C5-1-ST and C2-1-SC forming a gel network under isochoric conditions after MD equilibration. (*B*) Density distributions of cross-sections of the simulated gel networks from different C2 × C5 combinations. (*C* and *E*) Radial distribution function plots and the intensity of the first peak of different C2 × C5 combinations. (*D*) Fraction of empty grid voxels for the simulated gels. Voxel dimensions are x × y × z = 3,000 × 3,000 × 5. (*F*) Local structure of (*Left*) C2-1-SC x C5-1-ST and (*Right*) C2-10-SC × C5-10-ST systems.

To relate the rheological measurements to building block properties at the molecular level, we turned to molecular dynamics (MD) simulations. We developed a simplified protein model for the C2 × C5 systems with varying linker lengths using the HOOMD-Blue MD simulation toolkit (43), inspired by a coarse-grained model of DNA-grafted colloids (44). In this model, C2, C5, and SC are represented by rigid bodies of spheres connected by flexible (GGS)_n_ linkers and ST (*SI Appendix*, Fig. S2). To predict the porosity of the gel as a function of linker length, we calculated the local density fluctuations of the networks on a regular grid (Fig. 4 *A* and *B*). We found that the shortest linker gels had the highest predicted porosity, with the fraction of empty grid voxels decreasing as the linker length increased (Fig. 4*D*). This linker length-dependent porosity reflects the local clustering of the gels: The intensity of the first peak of the radial distribution function g(r) (Fig. 4 *C* and *E*) is stronger for the shorter linker gels indicating higher local clustering (45). The longer linker gels have radial distribution functions with less pronounced peaks, indicating more isotropic gel networks with fewer voids (Fig. 4*F*). These predictions are consistent with our experimental observations: shorter linkers may form more porous networks (as predicted), leading to lower stiffness.

#### Cytocompatability.

Having demonstrated that modular de novo protein-based hydrogels can be formed with tunable viscoelasticity from well-defined macromolecular precursors, we next investigated whether these systems would permit cytocompatible cell encapsulation and sustained 3D cell culture. As an initial test, 10T1/2 fibroblasts were suspended in a phosphate-buffered saline (PBS) solution of C2-5-SC prior to mixing with C5-5-ST. Following polymerization (10 µL droplets) at 37 °C, cells were maintained in culture for 24 h, with viability assessed. High viability was observed in each case (*SI Appendix*, Fig. S3), indicating the suitability of de novo protein-based hydrogels for 3D cell culture and its potential utility in tissue engineering.

### Noncovalent Protein Networks Exhibit Tunable Viscoelastic Fluid Properties Both In Vitro and In Cells.

**Fig. 5.**
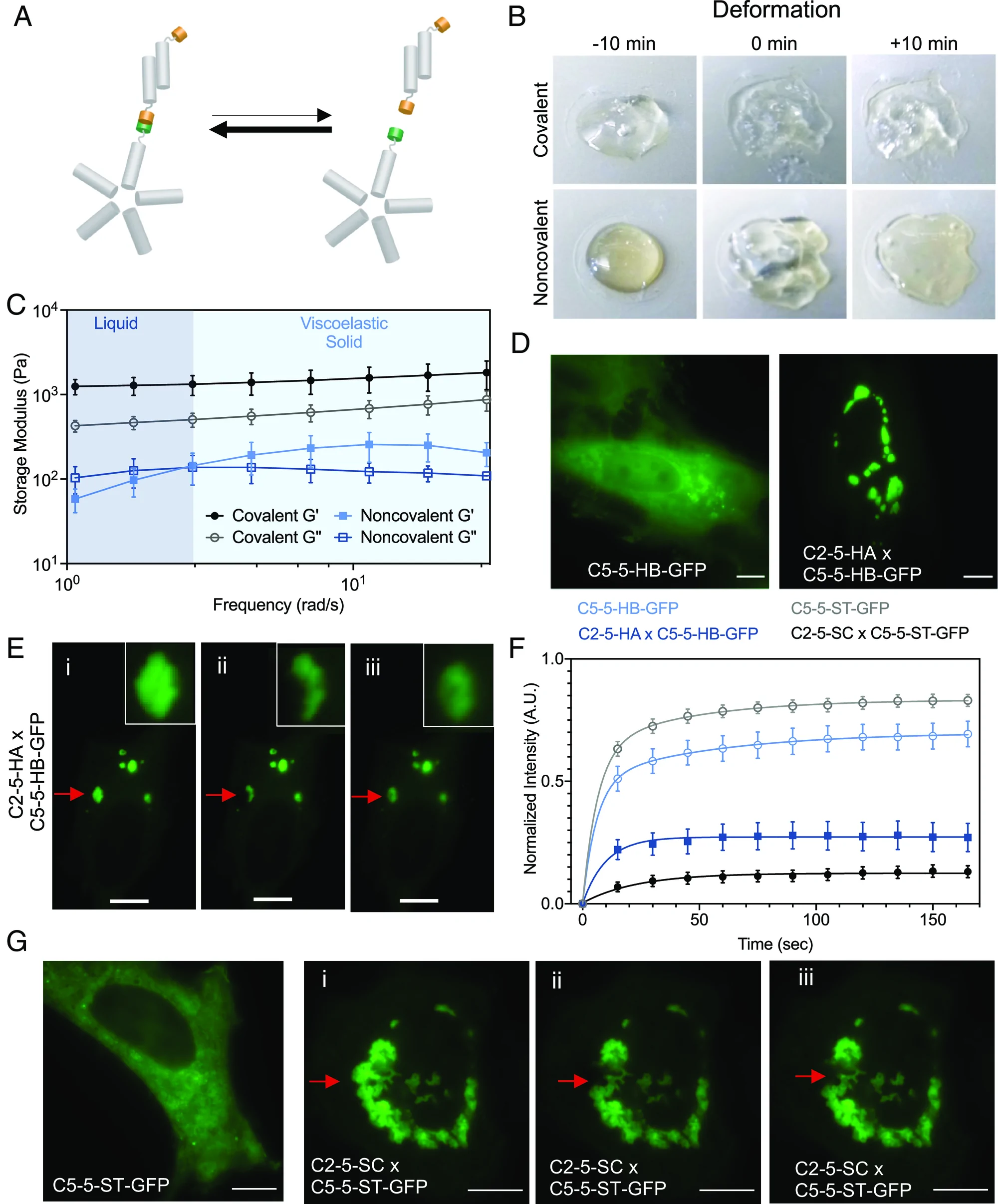
Viscoelastic fluid properties of the non-covalent assemblies. (*A*) Reversible formation of the LHD101 heterodimer within the C2-5-HA × C5-5-HB protein network. (*B*) Comparison of deformation of covalent vs. noncovalent hydrogels [Covalent: C2-5-SC × C5-5-ST; Noncovalent: C2-5-HA × C5-5-HB]. Pictures were taken 10 min before, at 0 min (at the point of deformation), and 10 min after deformation. (Scale bar, 4 mm.) (*C*) Rheometric studies (frequency sweep) show that the noncovalent assemblies behave as a viscoelastic liquid at resting or low frequency (G’<G”), but exhibit gel-like (viscoelastic solid) properties as the frequency is increased (G’>G’’). In contrast, covalent gels behave as viscoelastic gels throughout the frequency sweep (G’>G’’). (*D*) Designed non-covalent assemblies form in cells. C5-5-HB-GFP alone (*Left*) and coexpression of C2-5-HA and C5-5-HB-GFP (*Right*). (*E*) FRAP experiment showing intracellular noncovalent C2-5-HA × C5-5-HB-GFP assemblies as viscoelastic fluid droplets, similar to the extracellular experiment. i) before photobleaching, ii) immediately after photobleaching, and iii) after complete recovery (180 s). (*F*) Time-dependent fluorescence recovery plot of noncovalent assembly (C5-5-HB-GFP, control; and C2-5-HA × C5-5-HB-GFP), and covalent assembly (C5-5-ST-GFP, control; and C2-5-SC × C5-5-ST-GFP). Error bars indicate ± SEM. (*G*) Designed covalent gels form in cells. Expression of C5-5-ST-GFP in cells alone showed diffuse fluorescence, whereas coexpression of C5-5-ST-GFP and C2-5-SC led to the formation of stable intracellular punctae. FRAP experiments showing i) before photobleaching, ii) immediately after photobleaching, and iii) after 180 s. (Scale bar 10 µm.)

We next investigated the impact on material viscoelasticity when replacing the covalent interaction in the C2 × C5 hydrogel with a non-covalent, and thus reversible, interaction (Fig. 5*A*). We swapped the SC/ST with two chains of the de novo designed heterodimer LHD101, henceforth referred to as HA and HB, which are ~8.6 kDa each and interact with nanomolar binding affinity with on/off rates of ~2 × 10^6^ M^−1^ s^−1^ and ~4 × 10^−3^ s^−1^ respectively (40). C2-5-HA was mixed with C5-5-HB at equimolar ratios of HA and HB, and the mixture was inspected for viscoelastic material formation. The noncovalent mixture appeared droplet-like (Fig. 5 *B*, *Bottom Left*), in comparison to the analogous system with covalent SpyLigation-based interactions (Fig. 5 *B*, *Top Left*). Ten minutes following the application of a stress, the deformed noncovalent material reverted back to a droplet-like fluid, whereas the covalent materials remained as distorted gels (Fig. 5*B*). Further rheometric characterization showed that the noncovalent materials exhibited a lower storage modulus than loss modulus (G’<G”) at resting or low frequency indicating liquid-like properties; the storage moduli overtook the loss moduli (G’>G”) at higher frequencies. In contrast, the covalent gels all showed a higher storage modulus than the loss modulus (G’>G”) throughout the frequency sweep, indicating that these materials persisted throughout as solid gels (Fig. 5*C*). Given the noncovalent nature of the material crosslinks, we hypothesized that LHD101 gel networks would exhibit self-healing characteristics, which was verified by cyclic strain time sweep rheology (46): The LHD101 gel networks were capable of self-healing over multiple cycles of 500% strain (*SI Appendix*, Fig. S4).

This soft fluid behavior at resting or low applied frequency but gel-like behavior at a frequency above the crossover point is characteristic of hydrogels assembled through physical interaction (22, 47). We hypothesize that rapid equilibration between LHD101 heterodimers endows the material with fluid-like properties at the resting state, but once the stress is applied at a frequency faster than the on and off rates of the LHD101 A/B interaction, the intermolecular interaction between C2 and C5 (through HA and HB) are effectively locked, leading to gel-like properties (Fig. 5*A* and *SI Appendix*, Fig. S5). Once this strain is released, the material returns to the equilibrium fluid state (Fig. 5*B*, +10 min deformation).

The dynamic stress response and genetic encodability of the noncovalent network formed by C2 and C5 through the LHD101 heterodimer make such materials attractive for applications in synthetic biology. Recent studies have used peptide nanomaterials to make intracellular hydrogels (48), and pairs of naturally occurring proteins to make intracellular gels that mimic the function of RNA granules (49). We hypothesized that the analogous noncovalent interaction between the constituents (i.e., C2-5-HA and C5-5-HB) would lead to the intracellular formation of complex coacervates. To explore the behavior of the material in living mammalian cells, we co-expressed C2-5-HA and C5-5-HB-GFP in HeLa cells, where green fluorescent protein (GFP) was genetically fused to the latter to monitor assembly formation by fluorescent microscopy. When both network constituents were coexpressed, we observed intracellular droplet-like punctae, demonstrating intracellular networking and complex coacervation formation through noncovalent interactions (Fig. 5*D*). In contrast, the expression of C5-5-HB-GFP alone generated diffuse cytoplasmic fluorescence. Experiments in which both components were independently fluorescently tagged (i.e., C2-5-HA-mCherry and C5-5-HB-GFP) showed that droplet formation only occurred above threshold concentrations of both components (*SI Appendix*, Fig. S6).

To assess the fluid properties of the droplet, we performed fluorescence recovery after photobleaching (FRAP) experiments, in which a part of a droplet was photobleached and then allowed to recover fluorescence over time. The fluorescence recovery half-life time of the C2-5-HA × C5-5-HB-GFP droplet was 7.0 s (Fig. 5 *E* and *F* and Movie S1) indicating that the assemblies are not rigid hydrogels but are fluids, with their constituents reorganizing over time, similar to our rheological results. Photobleaching of control cells singly transfected with C5-5-HB-GFP showed a faster recovery half-life time of ~4.1 s, presumably due to free diffusion of the C5 assemblies when not crosslinked into a network (Fig. 5*F*, *SI Appendix*, Fig. S7, and Movie S2). The fluorescence intensity loss after recovery of the C2-5-HA × C5-5-HB-GFP droplet matched the fraction of the droplet remaining after bleaching, indicating that the reorganization of the C2-5-HA × C5-5-HB-GFP building blocks occurred within the droplet rather than exchange with the surrounding cytoplasm (*SI Appendix*, Fig. S8).

Analogous co-expression of the covalent hydrogel-forming components in mammalian cells resulted in stable intracellular gels. The expression of C5-5-ST-GFP alone generated diffuse cytoplasmic fluorescence (recovery half-life time ~2.17 s) (Fig. 5 *F* and *G* and Movie S4), whereas the co-expression of C2-5-SC × C5-5-ST-GFP resulted in an intracellular hydrogel that did not recover (or recovered extremely slowly) fluorescence after photobleaching (Fig. 5 *F* and *G* and Movie S3), suggesting a stiff gel. The bulk gels formed from purified C2-5-SC and C5-5-ST-GFP proteins exhibited similar FRAP characteristics, with only slight recovery of fluorescence after photobleaching (*SI Appendix*, Fig. S10).

### Designed Intracellular De Novo Protein Hydrogel Mechanics Correlate with Extracellular Gels.

**Fig. 6.**
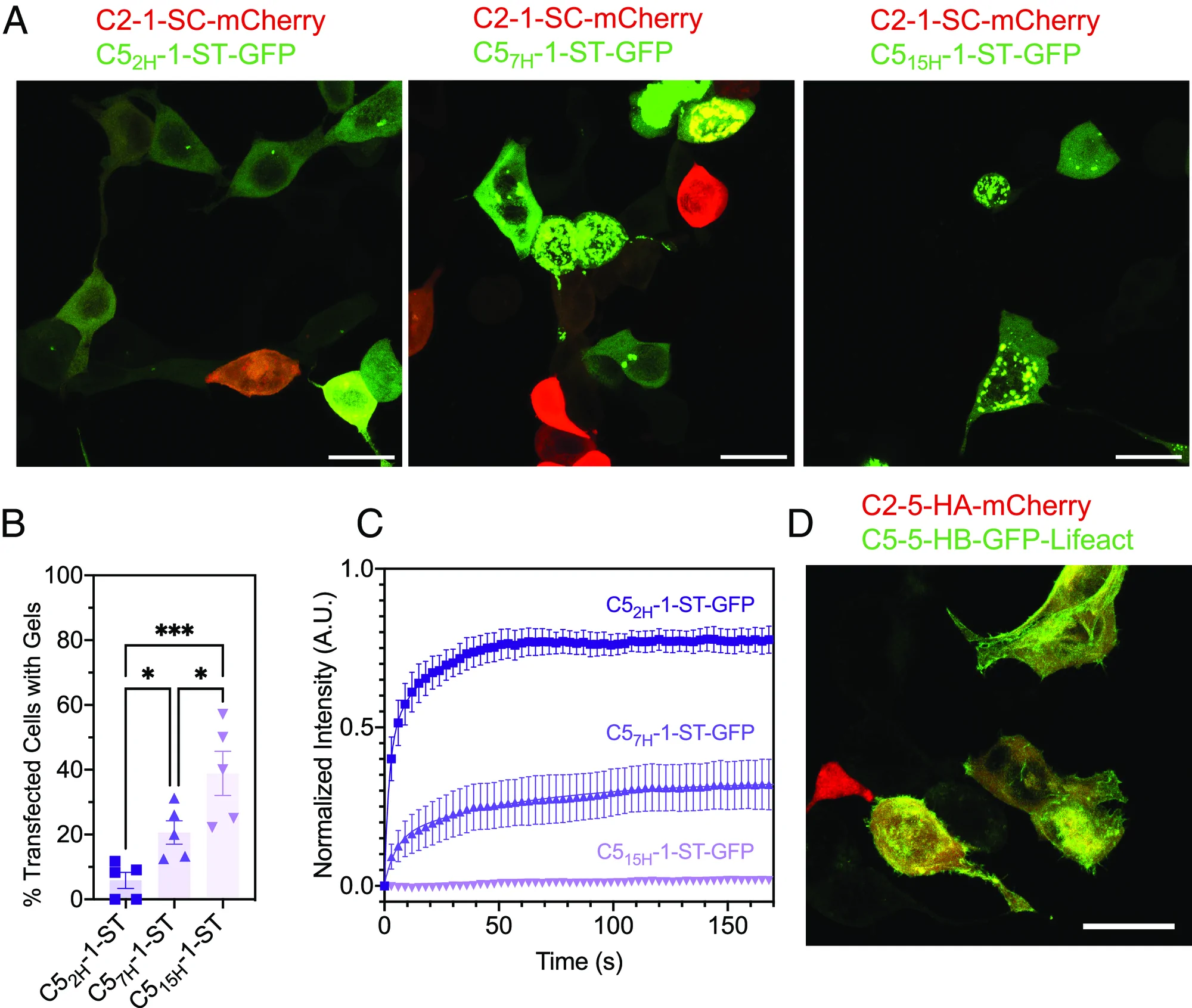
Intracellular gel mechanics correlates with properties of the corresponding extracellular gels. (*A*) Intracellular gel formation between C2-1-SC-mCherry and C5-ST-GFP with varied rigid arm length (2H, 7H, and 15H). C5_7H_-1-ST-GFP and C5_15H_-1-ST-GFP formed efficient gels with C2-1-SC-mCherry, whereas C5_2H_-1-ST-GFP failed to do so. The propensity of intracellular gel formation of these combinations (*B*) correlates with the stiffness of the corresponding extracellular gels (Fig. 2 *E* and *F*). (*C*) FRAP experiments showing the recovery of fluorescence after photobleaching the intracellular gels. (*D*) Targeting of intracellular gels to actin filaments through the fusion of a gel component to actin-targeting Lifeact sequence (C5-5-HB-GFP-Lifeact). (Scale bar, 20 µm.)

The C5-ST building blocks containing rigid arms formed covalent hydrogels with C2-SC, whose stiffness varied significantly with the change in rigid arm length (Fig. 2 *E* and *F*). Taking advantage of the distinct rheological properties offered by this series of hydrogel-forming building blocks, we co-expressed the constituents in mammalian cells to determine whether such hydrogels can be formed intracellularly and if their mechanical properties matched with the ex cellulo counterparts. Co-expression of C2-1-SC-mCherry with C5_2H_-1-ST-GFP in HEK293T cells resulted in diffuse fluorescence, whereas both C2-1-SC-mCherry × C5_7H_-1-ST-GFP and C2-1-SC-mCherry × C5_15H_-1-ST-GFP pairs showed droplet-like gel formation (Fig. 6*A*). As the formation of hydrogel droplets was contingent on the strong expression of the hydrogel components at appropriate stoichiometries, hydrogels did not form in all cells expressing either the C2-mCherry or C5-GFP constructs. Therefore, we measured the propensity of intracellular gel formation, defined as the percentage of cells expressing either GFP or mCherry where the fluorescent proteins had formed intracellular condensates. The propensity of gel formation increased in the order of 2H < 7H < 15H (rigid arm length) (Fig. 6*B*), exhibiting a strong positive correlation with rheologically measured bulk gel stiffnesses (Fig. 2*E*). FRAP studies of these droplet gels further reflected their mechanical strength: 15H gels did not recover their fluorescence (Movie S7) (recovery half-life time ~6,053 s), whereas 7H gels recovered up to about 30% of their original intensity (Movie S6) (recovery half-life time ~3.23 s). On the other hand, 2H assemblies failed to form any detectable gels and therefore recovered their fluorescence quickly (Movie S5) (recovery half-life time ~1.38 s). Collectively, these experiments indicate that the 15H system may have formed gels with no dynamic reorganization and exchange of the constituents, 7H formed gels with reorganization of the constituents to some extent, whereas 2H failed to form any gels and therefore resulted in free flowing of the constituents (Fig. 6*C* and *SI Appendix*, Fig. S9). To compare the above trends of intracellular gel mechanics with the ex cellulo counterpart, we performed FRAP studies on the bulk hydrogels formed by the same rigid arm system (2H, 7H, and 15H). Analogous to the intracellular system, 2H assemblies quickly recovered their fluorescence, whereas both the 7H and 15H gels exhibited little fluorescence recovery (*SI Appendix*, Fig. S10). In addition, the behavior of intracellular gel mechanics (measured through FRAP) mirrored trends in ex cellulo-formed gel mechanics (measured through elastic moduli) (Fig. 2 *E* and *F*).

We next investigated whether protein assemblies could be localized within cells through genetic appendage of binding domains. We designed a C5 construct fused with the actin-binding Lifeact peptide (50). The resultant construct (i.e., C5-5-HB-GFP-Lifeact) was co-expressed with C2-5-HA-mCherry in HEK293T cells. Fluorescence microscopy imaging showed the colocalization of the C2-5-HA-mCherry (red) with C5-5-HB-GFP-Lifeact (green) throughout the cytoplasmic actin filaments with visible punctate-like structures, indicating that these gels may be targeted to the cytoskeleton (Fig. 6*D*).

Intracellular liquid–liquid phase separated condensates have been generated from multivalent natural proteins with intrinsically disordered regions (51–54). Our de novo protein approach produces gels that resemble these condensates but goes beyond previous work by enabling finely tunable control over the fluid properties by varying the network chemistry and the structure of the components. This ability to finely modulate fluid properties could be useful for synthetic biology applications such as controlling enzyme sequestration, cell motility, and growth.

## Discussion

We have designed hydrogels from synthetic de novo protein building blocks with well-defined structures, valencies, and geometries connected in extended networks through either covalent or noncovalent interactions. Since the building blocks and interactions are well understood, our results provide a unique opportunity to relate the emergent macroscopic properties of hydrogels to the molecular and microscopic properties of the components. Full calculation of these properties would require extensive simulation efforts beyond the scope of this paper; here, instead, we have characterized the qualitative effects of varying building block topologies and association dynamics on hydrogel rheological properties. The dynamics of the component-component interaction driving hydrogel formation had a very large effect on the rheological properties. The covalent hydrogels formed using the SC/ST chemistry exhibited varied elastic moduli in a linker length (between the core and the interaction domain) and valency-dependent manner. In contrast, the non-covalent hydrogels formed using the reversibly associating designed LHD101 interaction exhibited dynamic viscoelastic properties in response to stress stimulus. Modulation of the secondary structure of gel constituents and of the network chemistry connecting them resulted in tunable biomaterials ranging in phase from fluid to gel and in storage moduli from soft to stiff, both in vitro and in cells.

There has been a growing interest in engineering extracellular matrices with defined stiffness for cellular differentiation and maintenance and intracellular synthetic membrane-less organelles for a wide variety of cell functions. Our ability to fine-tune the elastic properties of protein networks by modulating the length of both flexible and rigid linkers, the connection valency, and the dynamics of the interactions mediating hydrogel crosslinking, while keeping a core structured region unaltered, opens up avenues in synthetic biology and tissue engineering. The ability to genetically encode these networks extends the design space to within as well as between cells. Moreover, emerging protein–protein ligation chemistries offer opportunities to potentially trigger such de novo protein-based material formation, both within and outside of living cells (55). More generally, assembling hydrogels from custom-designed components provides a systematic approach to relate microscopic structure to macroscopic properties, and as these connections become better understood, to custom-design materials with desired viscoelasticity that can be formed both intra- and extracellularly.

## Methods

### Gene Preparation.

The amino acid sequences corresponding to respective oligomers (i.e., C2, C5, T33, I53, LHD101, and SpyCatcher/Tag) were derived from previous reports and placed into pET29b+ vector with necessary modification, and incorporation of (GGS)_n_ linker, etc. For the full list of sequences, please see *SI Appendix*. For two-component designs (T33 and I53), all designs were expressed bi-cistronically by appending an additional ribosome binding site in front of the second sequence, with only one of the components containing a 6xHis tag. Genes were synthesized by Integrated DNA Technologies.

### Protein Expression and Purification.

All genes in the pET29b+ vector were transformed into *E. coli* cells [BL21 Lemo21 (DE3)] for expression. Proteins were expressed using an auto-induction protocol at 37 °C for 16 to 24 h on a 500-mL scale. Cells were harvested by centrifugation at 4,000 × g for 10 min. Cell pellets were then resuspended in 25 to 30 mL lysis buffer (Tris-Buffered Saline (TBS), 25 mM Tris, 300 mM NaCl, pH8.0, 10 mM imidazole, 0.20 mg/mL DNase I) and sonicated for 2.5 min total on time at 80 to 90% power (10 s on/off) (QSonica). Lysates were then centrifuged at 14,000 × g for 30 min. Clarified lysates were then passed through Ni-NTA resin (QIAgen), washed with wash buffer (TBS, 25 mM Tris, 300 mM NaCl, pH 8.0, 40 mM imidazole), and then eluted with elution buffer (TBS, 25 mM Tris, 300 mM NaCl, pH 8.0, 500 mM imidazole). Note that each protein was expressed in 6 × 500 mL cultures, and the eluents were collected together to concentrate with a 10,000 m/w cutoff spin concentrator (Millipore) to make the final concentration at least 100 mg/mL.

### Hydrogel Preparation.

Covalent hydrogels were formed by mixing SpyCatcher (SC)-modified C2 with SpyTagged (ST) higher-order oligomer in a 1:1 molar ratio of SC:ST, keeping the total w/v concentration fixed at 10%. Most constructs formed hydrogel immediately after mixing, but the samples were kept for ~12 h for complete network formation. For visual inspection and gel photography, the mixtures were made in a cloning cylinder (4 mm inner diameter) in 40 microliter volume. The cylinder top was wrapped with a parafilm and kept at room temperature for 12 h, and digital photographs were taken after carefully removing the cylinder. Note that, for the C2-(GGS)_n_-SC protein series, we only have C2-1-SC and C2-5-SC, whereas, for the C5-(GGS)_n_-ST series, we have n = 1 (C5-1-ST), 5 (C5-5-ST), and 10 (C5-10-ST). We did not include a C2-10-SC analog for this study as its solubility was poor and it did not reach the required 100 mg/mL concentration mark.

Noncovalent hydrogels were made similar to the covalent at 20% w/v. For the deformation studies, C2-5-HA was mixed with C5-5-HB at an equimolar ratio of HA and HB on a parafilm sheet and allowed to crosslink fully for half an hour. Pictures were taken 10 min before deformation, instantly after deformation, and 10 min after deformation.

### Rheometry.

In situ rheology was conducted on an Anton Paar MCR 302 rheometer fitted with an 8mm parallel plate measurement attachment. Hydrogel droplets were formed at a volume of 30 µL and thickness of 0.5 mm on glass coverslips overnight in a humidified chamber prior to frequency sweep experiments. Gels on coverslips were then secured to the rheometer base plate for mechanical analysis. Frequency sweeps were conducted at a strain amplitude of 10% across frequencies from 0.1 to 500 rad/s unless otherwise stated. Linear viscoelasticity was verified in the strain amplitude domain by amplitude sweep rheology at frequencies in the linear viscoelastic region (1 rad/s for covalently crosslinked gels and 10 rad/s for noncovalently crosslinked gels) and amplitudes ranging from 1 to 100% strain. Cyclic strain time sweep experiments were conducted across 5 cycles of high strain (500% amplitude, 300 s) followed by a low-strain recovery period (1% amplitude, 1,800 s).

### Cell Encapsulation.

10T1/2 cells were mixed with gel precursors to form a 10% w/v (in 1× PBS) hydrogel containing 10^6^ cells/mL, of which 10 µL droplets were allowed to polymerize at 37 °C in a humidified 96-well tissue culture dish. After 60 min of polymerization, media was added. At the specified timepoints, gels were stained with a LIVE/DEAD Cell Imaging kit (Thermo Fisher) per the manufacturer’s instructions and imaged immediately on a Leica Stellaris 5 confocal microscope under 10× magnification. Viable and nonviable cells were counted using CellProfiler 4.0.

### Intracellular Assembly.

All genes (i.e., C5-5-HB-GFP, C5-5-HB-GFP-Lifeact, C2-5-HA, C2-5-HA-mCherry, C5-5-ST-GFP, C2-5-SC, C2-1-SC-mCherry, C5_2H_-1-ST-GFP, C5_7H_-1-ST-GFP, and C5_15H_-1-ST-GFP) were cloned into the pcDNA3.1+ mammalian expression vector, which were transfected into HeLa cells (ATCC CCL-2) or HEK293T [cultured in Dulbecco’s modified Eagle’s medium at 37 °C and 5% CO_2_] in the right combination. Briefly, cells were plated at 20,000 cells per well in Cellview cell culture slides (Greiner Bio-One ref 543079), then 24 h later, cells were transiently transfected at a concentration of 140 ng total DNA per well and 1 μg/μL PEI-MAX (Polyscience) mixed with Opti-MEM medium (Gibco). Transfected cells were incubated at 37 °C and 5% CO2 for 24 to 36 h before being imaged.

### Intracellular FRAP Imaging.

Bleaching movies were acquired with a commercial OMX-SR system (GE Healthcare) equipped with a Toptica diode 488 and 405 nm lasers and stage/objective heating set to 37 °C. Emission was collected on a PCO.edge sCMOS camera using an Olympus 60× 1.49NA ApoN oil immersion TIRF lens. Then, 1,024 × 1,024 images (pixel size 6.5 μm) were captured with no binning. The acquisition was controlled with AcquireSR Acquisition control software. Photobleaching was carried out by acquiring a reference image followed by bleaching of a spot using a 600 ms pulse from a 405-nm laser. Recovery was monitored by imaging every 3 (rigid arm series) or 15 s for 3 min.

For FRAP analysis, FRAP data were plotted and fit to a single exponential association model using GraphPad Prism Version 9.3.1. Three ROIs of the same size were drawn on each image to measure a) FRAP spot intensity (I_FRAP_), b) a reference spot for photobleaching correction (I_Ref_), and c) a baseline spot outside the cell for background correction (I_Base_). The baseline intensity was first subtracted from both I_FRAP_ and I_Ref_ to correct for image background (56).

I_FRAP Corr_ = I_FRAP_ − I_Base_

I_Ref Corr_ = I_Ref_ − I_Base_

Next, correction for bleaching and normalization of intensities was performed using the following equation:

I_Norm_ = (I_Ref_pre_/I_Ref_corr(t)_) * (I_FRAP_corr(t)_/I_FRAP_pre_),

where I_Ref_pre_ and I_FRAP_pre_ are the pre-bleaching background-corrected intensities and I_Ref_corr(t)_ and I_FRAP_corr(t)_ are the background-corrected intensities at each time point.

Finally, all traces were averaged and fitted to exponential association equations giving the half-time of fluorescence recovery and immobile fraction.

### FRAP Analysis of Bulk Hydrogel.

Hydrogel components were mixed to make gels of 10% w/v and spotted onto an 18-mm glass coverslip and adhered to a microscope slide via a SecureSeal imaging spacer, 9 mm diameter. Samples were allowed to rest for 60 min to form hydrogel fully prior to imaging.

In vitro FRAP experiments were carried out on a Leica SP8X confocal microscope equipped with a motorized XY stage and a 25× water immersion objective. Pre- and post-bleaching acquisition images were acquired using a 476-nm argon fixed wavelength laser line using adaptive focus control for maintaining focus during live imaging and a PMT amplification detector to record output. Bleaching was carried out using 100% laser power of the 476 nm laser and 10 successive scans over the bleaching area to ensure efficient bleaching of each spot. Post-bleaching images were recorded every 5 s for 15 min to ensure complete recovery in all conditions.

FRAP analysis began by using the Fast4DReg plugin in Fiji (ImageJ) (https://github.com/guijacquemet/Fast4DReg) to correct for drift in the collected images. This was followed by a custom Fiji script used to measure intensity in bleached spots as well as a reference spot outside the bleaching area. Intensity measurements were then normalized to the pre-bleach intensity of each scan and corrected for photobleaching during acquisition using the reference area. Recovery traces were fit to double exponential equations in GraphPad Prism.

### Molecular Dynamics.

Simulations were conducted using MD implemented in the HOOMD-blue particle simulation package (https://github.com/glotzerlab/hoomd-blue) (43). In the simulation model, C5 is represented as a rigid body of spheres with five-fold symmetry, which is connected by a flexible GGS linkers and SpyTag (*SI Appendix*, Fig. S2 and Table S1). The beads of the flexible linkers are bonded each other by a harmonic potential, $V_{\mathrm{bond}}=\left(\frac{1}{2}\right)k{\left(r-r_{0}\right)}^{2}$, where $k=300\;\varepsilon /\sigma^{2}$ and $r_{0}=1.0\;\sigma$. Here, *r* is the distance between the centers of beads of the GGS linker, *σ* is the distance unit, and *ε* is the energy unit in simulation. Similarly, C2 is represented as a rigid body of spheres that are linearly connected, and the rigid body of C2 is connected by flexible GGS linkers and SpyCatcher body (*SI Appendix*, Fig. S2*B*). The SpyCatcher body is represented as a rigid body of spheres that are connected in a cylindrical shape, and there are SpyCatcher patches at the center of the cylinder (*SI Appendix*, Fig. S2 *B* and *C*), and the patches interact with SpyTag of C5 through an attractive Gaussian potential, $V_{\mathrm{Gauss}}\left(r\right)=\varepsilon_{\mathrm{Gauss}}exp\left[-1/2{\left(r/\sigma_{\mathrm{Gauss}}\right)}^{2}\right]$, where $\varepsilon_{\mathrm{Gauss}}=2.0\;\varepsilon$, $\sigma_{\mathrm{Gauss}}=0.6\;\sigma$, and *r* is the distance between the center of a bead of SpyTag and the center of a SpyCatcher.patch. All beads interact with each other (except SpyTag – SpyCatch patch pairs) via a purely repulsive WCA potential (57) to avoid overlap:

$$\begin{array}{c} V_{\mathrm{WCA}}\left(r\right)=\left\{\begin{array}{ll} 4\varepsilon_{\text{WCA}}\left[{\left(\frac{\sigma_{\text{ij}}}{r}\right)}^{12}-{\left(\frac{\sigma_{\text{ij}}}{r}\right)}^{6}\right]-4\varepsilon_{\text{WCA}}\left[{\left(\frac{\sigma_{\text{ij}}}{r_{\text{cut}}}\right)}^{12}-{\left(\frac{\sigma_{\text{ij}}}{r_{\text{cut}}}\right)}^{6}\right], & r<r_{\text{cut}}\\ 0 & r\geq r_{\text{cut}} \end{array}\right., \end{array}$$

where $\varepsilon_{\text{WCA}}=0.1\;\varepsilon$, $\sigma_{\text{ij}}=r_{i}+r_{j}$, $r_{\text{cut}}=\sigma_{\text{ij}}\times 2^{1/6}$, *i* and *j* are the type of beads that are interacting with, and *r_i_* is the radius of *i* bead listed in *SI Appendix*, Table S1. We initialized system of $N_{C5}=400$ and $N_{C2}=1,000$ in a periodic box with a very dilute condition, $\phi =\left(V_{C5}+V_{C2}\right)/V_{\text{Box}}\sim 6.2\times 10^{-4}$, where $V_{C5}$, $V_{C2}$, and $V_{\text{Box}}$ are the volume of C5, C2, and simulation box, respectively. The $V_{C5}$ and $V_{C2}$ do not include the volume of the GGS linker to have consistency throughout all systems. The initial positions of C5 and C2 are randomly assigned within the simulation box without making overlap. Various C2-*n* × C5-*m* combinations were used for each system, where *n* and *m* are the length of the GGS linker and *n* = 1, 3, 5, 10 and *m* = 1, 3, 5, 10. We compressed the system to ϕ=0.023 for 10^5^ MD timesteps and thermalized for 5 × 10^7^ MD timesteps in NVT ensemble ($T^{*}=0.2kT/\varepsilon$), where T* is the reduced temperature and each MD timestep is 0.002 for all systems. The density distribution of the simulated hydrogels (Fig. 4*B*) was computed using GaussianDensity module implemented in freud python package (58). The coordinates of every C5 body, C2 body, and SpyCatcher body were used for the density calculation. The g(r) (Fig. 4*C*) of the system was computed based on the center of mass of C5 and C2.

## Supplementary Material

Appendix 01 (PDF)

Movie S1. Fluorescence recovery after photobleaching (FRAP) imaging of the noncovalent C2-5-HA x C5-5-HB-GFP droplet in HeLa cell.

Movie S2. FRAP imaging of C5-5-HB-GFP alone in HeLa cell.

Movie S3. FRAP imaging of covalent C2-5-SC x C5-5-ST-GFP gel in HeLa cell.

Movie S4. FRAP imaging of C5-5-ST-GFP alone in HeLa cell.

Movie S5. FRAP imaging of covalent C2-1-SC x C5_2H_-1-ST-GFP in Hela cell.

Movie S6. FRAP imaging of covalent C2-1-SC x C5_7H_-1-ST-GFP in Hela cell.

Movie S7. FRAP imaging of covalent C2-1-SC x C5_15H_-1-ST-GFP in Hela cell.

## Data Availability

All study data are included in the article and/or supporting information.

## References

[1] Y. S. Zhang, A. Khademhosseini, Advances in engineering hydrogels. Science **356**, eaaf3627 (2017). 10.1126/science.aaf3627 PMC5841082 28473537

[2] M. P. Lutolf, P. M. Gilbert, H. M. Blau, Designing materials to direct stem-cell fate. Nature **462**, 433–441 (2009). 10.1038/nature08602 PMC2908011 19940913

[3] O. Chaudhuri , Hydrogels with tunable stress relaxation regulate stem cell fate and activity. Nat. Mater. **15**, 326–334 (2016). 10.1038/nmat4489 PMC4767627 26618884

[4] N. Gjorevski , Designer matrices for intestinal stem cell and organoid culture. Nature **539**, 560–564 (2016). 10.1038/nature20168 27851739

[5] D. Seliktar, Designing cell-compatible hydrogels for biomedical applications. Science **336**, 1124–1128 (2012). 10.1126/science.1214804 22654050

[6] C. A. DeForest, K. S. Anseth, Advances in bioactive hydrogels to probe and direct cell fate. Annu. Rev. Chem. Biomol. Eng. **3**, 421–444 (2012). 10.1146/annurev-chembioeng-062011-080945 22524507

[7] J. A. Shadish, G. M. Benuska, C. A. DeForest, Bioactive site-specifically modified proteins for 4D patterning of gel biomaterials. Nat. Mater. **18**, 1005–1014 (2019). 10.1038/s41563-019-0367-7 PMC6706293 31110347

[8] B. A. Badeau, M. P. Comerford, C. K. Arakawa, J. A. Shadish, C. A. DeForest, Engineered modular biomaterial logic gates for environmentally triggered therapeutic delivery. Nat. Chem. **10**, 251–258 (2018). 10.1038/nchem.2917 PMC5822735 29461528

[9] M. Hörner , Phytochrome-based extracellular matrix with reversibly tunable mechanical properties. Adv. Mater. **31**, e1806727 (2019). 10.1002/adma.201806727 30687975

[10] D. Wu , Polymers with controlled assembly and rigidity made with click-functional peptide bundles. Nature **574**, 658–662 (2019). 10.1038/s41586-019-1683-4 31666724

[11] S. R. Caliari, J. A. Burdick, A practical guide to hydrogels for cell culture. Nat. Methods **13**, 405–414 (2016). 10.1038/nmeth.3839 PMC5800304 27123816

[12] E. Prince, E. Kumacheva, Design and applications of man-made biomimetic fibrillar hydrogels. Nat. Rev. Mater. **4**, 99–115 (2019).

[13] R. Silva, B. Fabry, A. R. Boccaccini, Fibrous protein-based hydrogels for cell encapsulation. Biomaterials **35**, 6727–6738 (2014). 10.1016/j.biomaterials.2014.04.078 24836951

[14] W. A. Petka, J. L. Harden, K. P. McGrath, D. Wirtz, D. A. Tirrell, Reversible hydrogels from self-assembling artificial proteins. Science **281**, 389–392 (1998). 10.1126/science.281.5375.389 9665877

[15] W. Shen, R. G. H. Lammertink, J. K. Sakata, J. A. Kornfield, D. A. Tirrell, Assembly of an artificial protein hydrogel through leucine zipper aggregation and disulfide bond formation. Macromolecules **38**, 3909–3916 (2005).

[16] C. T. S. Wong Po Foo, J. S. Lee, W. Mulyasasmita, A. Parisi-Amon, S. C. Heilshorn, Two-component protein-engineered physical hydrogels for cell encapsulation. Proc. Natl. Acad. Sci. U.S.A. **106**, 22067–22072 (2009). 10.1073/pnas.0904851106 PMC2791665 20007785

[17] S. Banta, I. R. Wheeldon, M. Blenner, Protein engineering in the development of functional hydrogels. Annu. Rev. Biomed. Eng. **12**, 167–186 (2010). 10.1146/annurev-bioeng-070909-105334 20420519

[18] H. Li, N. Kong, B. Laver, J. Liu, Hydrogels constructed from engineered proteins. Small **12**, 973–987 (2016). 10.1002/smll.201502429 26707834

[19] F. Sun, W.-B. Zhang, A. Mahdavi, F. H. Arnold, D. A. Tirrell, Synthesis of bioactive protein hydrogels by genetically encoded SpyTag-SpyCatcher chemistry. Proc. Natl. Acad. Sci. U.S.A. **111**, 11269–11274 (2014). 10.1073/pnas.1401291111 PMC4128157 25049400

[20] N. Kong, Q. Peng, H. Li, Rationally designed dynamic protein hydrogels with reversibly tunable mechanical properties. Adv. Funct. Mater. **24**, 7310–7317 (2014).

[21] J. Wu , Rationally designed synthetic protein hydrogels with predictable mechanical properties. Nat. Commun. **9**, 620 (2018). 10.1038/s41467-018-02917-6 PMC5809592 29434258

[22] L. J. Dooling, M. E. Buck, W.-B. Zhang, D. A. Tirrell, Programming molecular association and viscoelastic behavior in protein networks. Adv. Mater. **28**, 4651–4657 (2016). 10.1002/adma.201506216 27061171

[23] P.-S. Huang, S. E. Boyken, D. Baker, The coming of age of de novo protein design. Nature **537**, 320–327 (2016). 10.1038/nature19946 27629638

[24] A. Leaver-Fay , ROSETTA3: An object-oriented software suite for the simulation and design of macromolecules. Methods Enzymol. **487**, 545–574 (2011). 10.1016/B978-0-12-381270-4.00019-6 PMC4083816 21187238

[25] J. A. Fallas , Computational design of self-assembling cyclic protein homo-oligomers. Nat. Chem. **9**, 353–360 (2017). 10.1038/nchem.2673 PMC5367466 28338692

[26] Y. Hsia , Design of multi-scale protein complexes by hierarchical building block fusion. Nat. Commun. **12**, 2294 (2021). 10.1038/s41467-021-22276-z PMC8052403 33863889

[27] N. P. King , Accurate design of co-assembling multi-component protein nanomaterials. Nature **510**, 103–108 (2014). 10.1038/nature13404 PMC4137318 24870237

[28] N. P. King , Computational design of self-assembling protein nanomaterials with atomic level accuracy. Science **336**, 1171–1174 (2012). 10.1126/science.1219364 PMC4138882 22654060

[29] Y. Hsia , Design of a hyperstable 60-subunit protein dodecahedron. [corrected]. Nature **535**, 136–139 (2016). 10.1038/nature18010 PMC4945409 27309817

[30] J. B. Bale , Accurate design of megadalton-scale two-component icosahedral protein complexes. Science **353**, 389–394 (2016), 10.1126/science.aaf8818. PMC5485857 27463675

[31] H. Shen , De novo design of self-assembling helical protein filaments. Science **362**, 705–709 (2018), 10.1126/science.aau3775. PMC6637945 30409885

[32] S. Gonen, F. DiMaio, T. Gonen, D. Baker, Design of ordered two-dimensional arrays mediated by noncovalent protein-protein interfaces. Science **348**, 1365–1368 (2015). 10.1126/science.aaa9897 26089516

[33] C. J. Lanci , Computational design of a protein crystal. Proc. Natl. Acad. Sci. U.S.A. **109**, 7304–7309 (2012). 10.1073/pnas.1112595109 PMC3358839 22538812

[34] J. Marcandalli , Induction of potent neutralizing antibody responses by a designed protein nanoparticle vaccine for respiratory syncytial virus. Cell **176**, 1420–1431.e17 (2019). 10.1016/j.cell.2019.01.046 PMC6424820 30849373

[35] A. C. Walls , Elicitation of potent neutralizing antibody responses by designed protein nanoparticle vaccines for SARS-CoV-2. Cell **183**, 1367–1382.e17 (2020). 10.1016/j.cell.2020.10.043 PMC7604136 33160446

[36] R. Divine , Designed proteins assemble antibodies into modular nanocages. Science **372**, eabd9994 (2021). 10.1126/science.abd9994 PMC8592034 33795432

[37] A. Courbet , Computational design of mechanically coupled axle-rotor protein assemblies. Science **376**, 383–390 (2022). 10.1126/science.abm1183 PMC10712554 35446645

[38] G. Ueda , Tailored design of protein nanoparticle scaffolds for multivalent presentation of viral glycoprotein antigens. eLife **9**, e57659 (2020). 10.7554/eLife.57659 PMC7402677 32748788

[39] B. Zakeri , Peptide tag forming a rapid covalent bond to a protein, through engineering a bacterial adhesin. Proc. Natl. Acad. Sci. U.S.A. **109**, E690–E697 (2012). 10.1073/pnas.1115485109 PMC3311370 22366317

[40] D. D. Sahtoe , Reconfigurable asymmetric protein assemblies through implicit negative design. Science **375**, eabj7662 (2022). 10.1126/science.abj7662 PMC9881579 35050655

[41] T. J. Brunette , Exploring the repeat protein universe through computational protein design. Nature **528**, 580–584 (2015). 10.1038/nature16162 PMC4845728 26675729

[42] A. Ambrogelly, The different colors of mAbs in solution. Antibodies (Basel) **10**, 21 (2021). 10.3390/antib10020021 PMC8161444 34073775

[43] J. A. Anderson, J. Glaser, S. C. Glotzer, HOOMD-blue: A Python package for high-performance molecular dynamics and hard particle Monte Carlo simulations. Comput. Mater. Sci. **173**, 109363 (2020).

[44] T. I. N. G. Li, R. Sknepnek, R. J. Macfarlane, C. A. Mirkin, M. Olvera de la Cruz, Modeling the crystallization of spherical nucleic acid nanoparticle conjugates with molecular dynamics simulations. Nano Lett. **12**, 2509–2514 (2012). 10.1021/nl300679e 22458569

[45] E. J. Meijer, D. Frenkel, Computer simulation of polymer-induced clustering of colloids. Phys. Rev. Lett. **67**, 1110–1113 (1991). 10.1103/PhysRevLett.67.1110 10045078

[46] M. H. Chen , Methods to assess shear-thinning hydrogels for application As injectable biomaterials. ACS Biomater. Sci. Eng. **3**, 3146–3160 (2017). 10.1021/acsbiomaterials.7b00734 PMC5727472 29250593

[47] S. Tang, M. Wang, B. D. Olsen, Anomalous self-diffusion and sticky rouse dynamics in associative protein hydrogels. J. Am. Chem. Soc. **137**, 3946–3957 (2015). 10.1021/jacs.5b00722 25764061

[48] D. T. Tomares, S. Whitlock, M. Mann, E. DiBernardo, W. S. Childers, Repurposing peptide nanomaterials as synthetic biomolecular condensates in bacteria. ACS Synth. Biol. **11**, 2154–2162 (2022). 10.1021/acssynbio.2c00078 35658421

[49] H. Nakamura , Intracellular production of hydrogels and synthetic RNA granules by multivalent molecular interactions. Nat. Mater. **17**, 79–89 (2018). 10.1038/nmat5006 PMC5916848 29115293

[50] B. J. Belin, L. M. Goins, R. D. Mullins, Comparative analysis of tools for live cell imaging of actin network architecture. Bioarchitecture **4**, 189–202 (2014). 10.1080/19490992.2014.1047714 PMC4914014 26317264

[51] P. Li , Phase transitions in the assembly of multi-valent signaling proteins. Nature **483**, 336–340 (2012). 10.1038/nature10879 PMC3343696 22398450

[52] M. Dzuricky, B. A. Rogers, A. Shahid, P. S. Cremer, A. Chilkoti, De novo engineering of intracellular condensates using artificial disordered proteins. Nat. Chem. **12**, 814–825 (2020). 10.1038/s41557-020-0511-7 PMC8281385 32747754

[53] Y. Dai, L. You, A. Chilkoti, Engineering synthetic biomolecular condensates. Nat. Rev. Bioeng., 10.1038/s44222-023-00052-6 (2023). PMC10107566 37359769

[54] A. A. Hyman, C. A. Weber, F. Jülicher, Liquid-liquid phase separation in biology. Annu. Rev. Cell Dev. Biol. **30**, 39–58 (2014). 10.1146/annurev-cellbio-100913-013325 25288112

[55] E. R. Ruskowitz , Spatiotemporal functional assembly of split protein pairs through a light-activated SpyLigation. Nat. Chem. **15**, 694–704 (2023). 10.1038/s41557-023-01152-x PMC10164143 37069270

[56] R. D. Phair, S. A. Gorski, T. Misteli, “Measurement of dynamic protein binding to chromatin in vivo, using photobleaching microscopy” in Chromatin and Chromatin Remodeling Enzymes, Part A (Methods in Enzymology, Academic Press, 2003), pp. 393–414. 10.1016/s0076-6879(03)75025-3 14870680

[57] D. Chandler, J. D. Weeks, H. C. Andersen, Van der waals picture of liquids, solids, and phase transformations. Science **220**, 787–794 (1983). 10.1126/science.220.4599.787 17834156

[58] V. Ramasubramani , freud: A software suite for high throughput analysis of particle simulation data. Comput. Phys. Commun. **254**, 107275 (2020).

