# Supplementary material for: De novo design of modular protein hydrogels with programmable intra- and extracellular viscoelasticity
Source: Proc Natl Acad Sci U S A. 2024 Jan 30;121(6):e2309457121. doi: 10.1073/pnas.2309457121 (PMC10861882; doi:10.1073/pnas.2309457121)
Supplement: Supplementary file 1 [file pnas.2309457121.sapp.pdf]

Supplementary Information

De novo design of modular protein hydrogels with programmable intra- and extracellular viscoelasticity

Rubul Mout[1,2,3,#,*], Ross C. Bretherton[4,5,6,7,8,#], Justin Decarreau[1,2], Sangmin Lee[1,2], Nicole Gregorio[4,5,6,7,8], Natasha I. Edman[1,2,9,10], Maggie Ahlrichs[1,2], Yang Hsia[1,2], Danny D. Sahtoe[1,2,11], George Ueda[1,2], Alee Sharma[12], Rebecca Schulman[13,14], Cole A. DeForest[2,4,5,6,7,8*], David Baker[1,2,11*]

[1]Department of Biochemistry, University of Washington, Seattle, WA 98195
[2]Institute for Protein Design, University of Washington, Seattle, WA 98195
[3]Stem Cell Program at Boston Children's Hospital, Harvard Medical School, Boston, MA 02115
[4]Department of Bioengineering, University of Washington, Seattle, WA 98195
[5]Department of Chemical Engineering, University of Washington, Seattle, WA 98195
[6]Institute for Stem Cell and Regenerative Medicine, University of Washington, Seattle, WA 98195
[7]Department of Chemistry, University of Washington, Seattle, WA 98195
[8]Molecular Engineering & Sciences Institute, University of Washington, Seattle, WA 98195
[9]Molecular and Cellular Biology Graduate Program, University of Washington, Seattle, WA 98195
[10]Medical Scientist Training Program, University of Washington, Seattle, WA 98195
[11]Howard Hughes Medical Institute, University of Washington, Seattle, WA 98195
[12]College of Professional Studies, Northeastern University, Boston, MA 02115
[13]Department of Chemical and Biomolecular Engineering, Johns Hopkins University, Baltimore, MD 21218
[14]Department of Computer Science, Johns Hopkins University, Baltimore, MD 21218
[#]These authors contributed equally: Rubul Mout, Ross Bretherton
*Corresponding authors. E-mail: Rubul Mout: rubul.mout@childrens.harvard.edu; Cole A. DeForest: profcole@uw.edu; David Baker: dabaker@uw.edu

Supplementary Movie Legends

Supplementary movie 1: Fluorescence recovery after photobleaching (FRAP) imaging of the noncovalent C2-5-HA x C5-5-HB-GFP droplet in HeLa cell.
Supplementary movie 2: FRAP imaging of C5-5-HB-GFP alone in HeLa cell.
Supplementary movie 3: FRAP imaging of covalent C2-5-SC x C5-5-ST-GFP gel in HeLa cell.
Supplementary movie 4: FRAP imaging of C5-5-ST-GFP alone in HeLa cell.
Supplementary movie 5: FRAP imaging of covalent C2-1-SC x $C5_{2H}$-1-ST-GFP in Hela cell.
Supplementary movie 6: FRAP imaging of covalent C2-1-SC x $C5_{7H}$-1-ST-GFP in Hela cell.
Supplementary movie 7: FRAP imaging of covalent C2-1-SC x $C5_{15H}$-1-ST-GFP in Hela cell.

Supplementary Figures

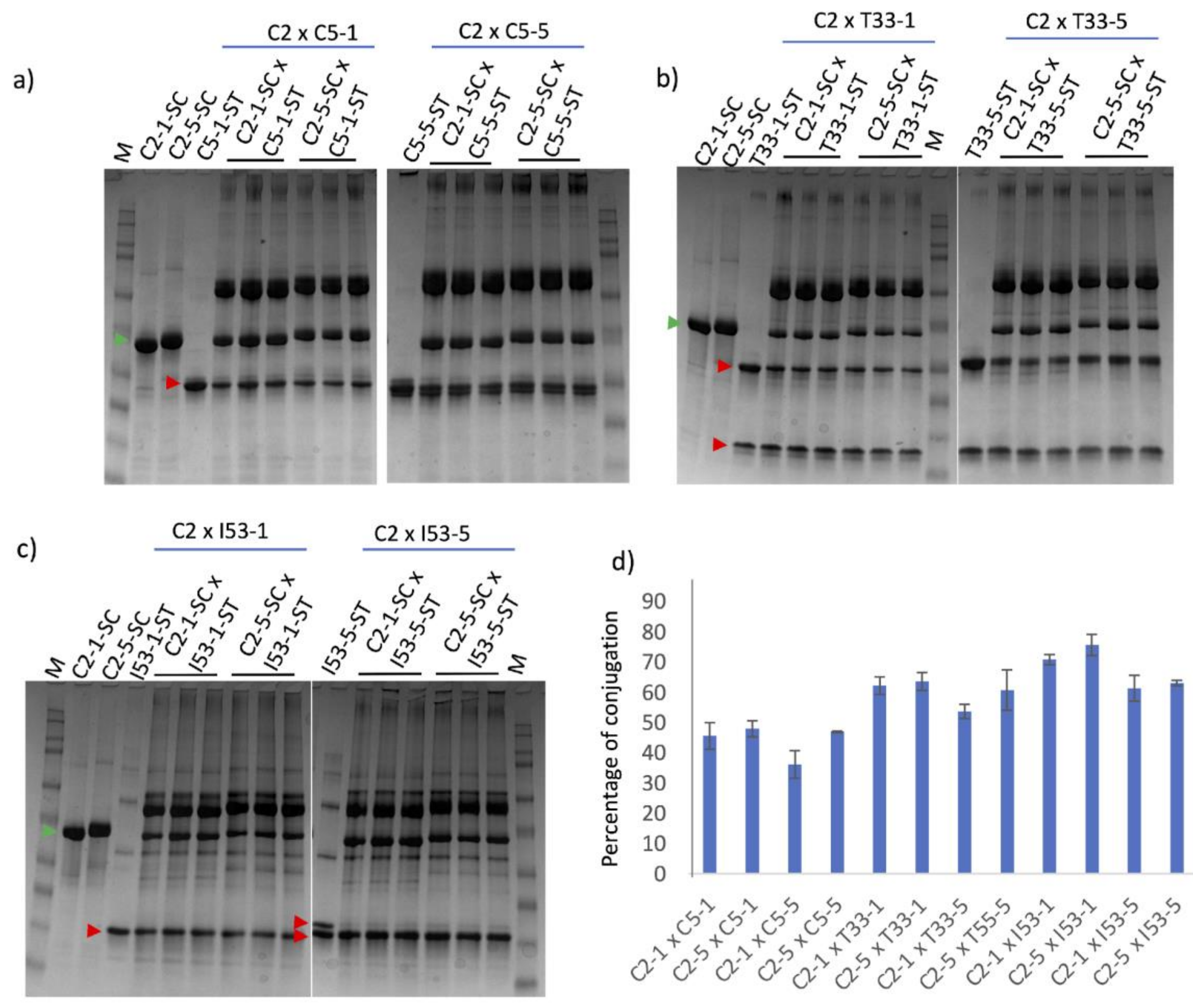

Figure S1. SDS-PAGE gel showing covalent crosslinking between SpyCatcher-modified C2 (green arrow) and SpyTagged higher-order oligomers (red arrow). After hydrogel formation between the respective constructs, samples were dissolved in denaturing (SDS) running buffer and boiled before running on a SDS-PAGE. Results show that upon covalent network formation through SpyLigation, the conjugated oligomers shift to higher position on the gel. a) C2 x C5; b) C2 x T33; and c) C2 x I53 hydrogel systems. Note that T33 and I53 oligomers are constituted of two components and hence two bands are observed in the gel (marked with red arrows). However, in I53-1-ST, both the components have identical sizes and the two bands are not distinguishable. Hydrogels of each formulation (*i.e.*, C2 x C5, C2 x T33, C2 x I53) were run in

triplicate (a, b, c). M: standard protein ladder. d) Quantitative estimation of hydrogel network formation (conjugation efficiency) between the components in each hydrogel. Briefly, the SDS-PAGE images in a, b, and c were subjected to image analysis to quantify the percentage conjugation efficiency. Percentage of unreacted C2-1 or C2-5 in each gel was estimated using ImageJ to determine the percentage conjugation. Results show that the conjugation efficiency varies between ~35% to ~75% among the different constructs.

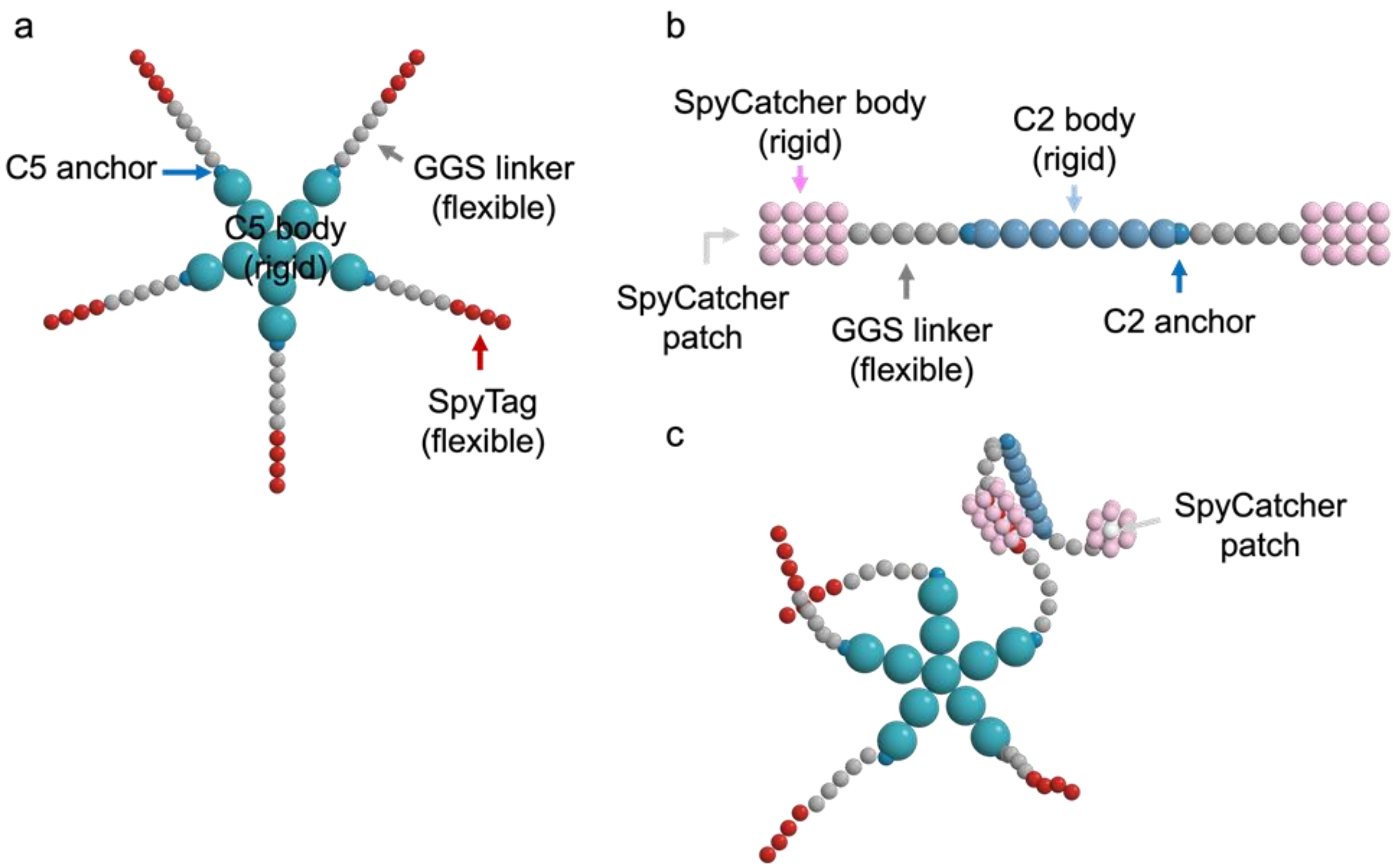

Figure S2. MD simulation model with representative examples C5-5-ST and C2-5-SC. (**a**) C5 consists of C5 body, C5 anchor, GGS linker, and SpyTag, and their coordinates are listed in Table S1. (**b**) C2 consists of C2 body, C2 anchor, GGS linker, SpyCatcher body, and SpyCatcher patch. (**c**) An attractive potential is applied between SpyTag and SpyCatcher patch so that the SpyTag can go into the SpyCatcher body.

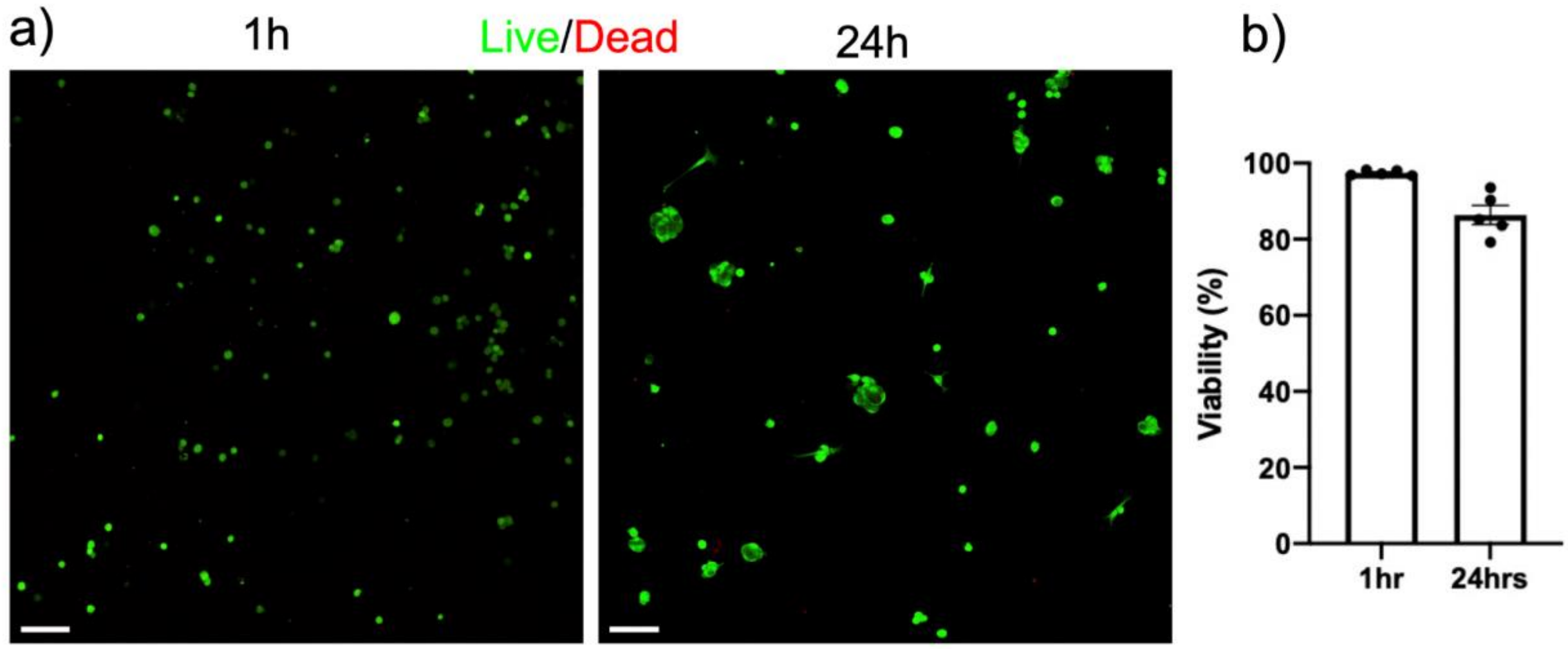

Figure S3: Cell encapsulation of 10T1/2 fibroblast cells in C2-5-SC x C5-5-ST gels. a) Cells were encapsulated and grown for 1 and 24h. Cell viability was assessed by LIVE/DEAD staining and confocal imaging. b) Percentage of cell viability was quantified by CellProfiler. Scale bar 20 µm.

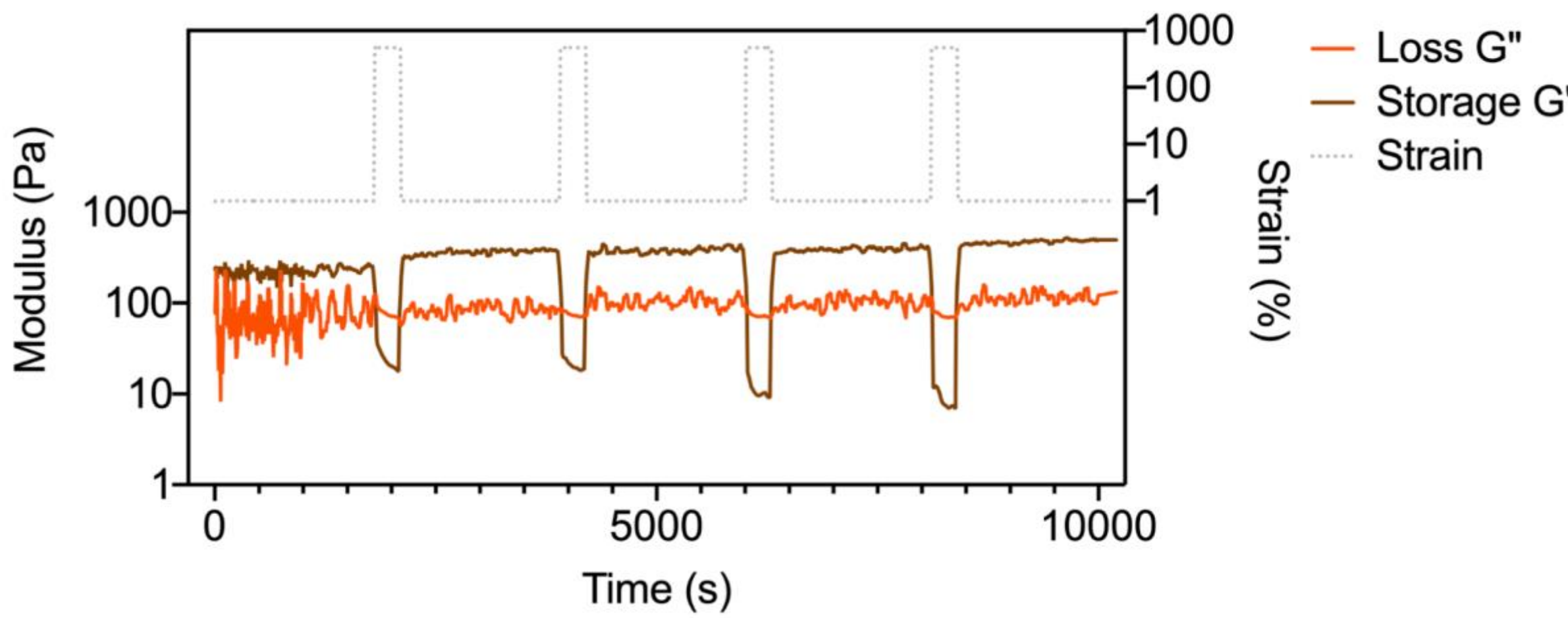

Figure S4. Self-healing properties of C2-5-HA x C5-5-HB noncovalent protein networks mediated by LHD101 heterodimer. The gel networks were capable of self-healing over multiple cycles of 500% strain.

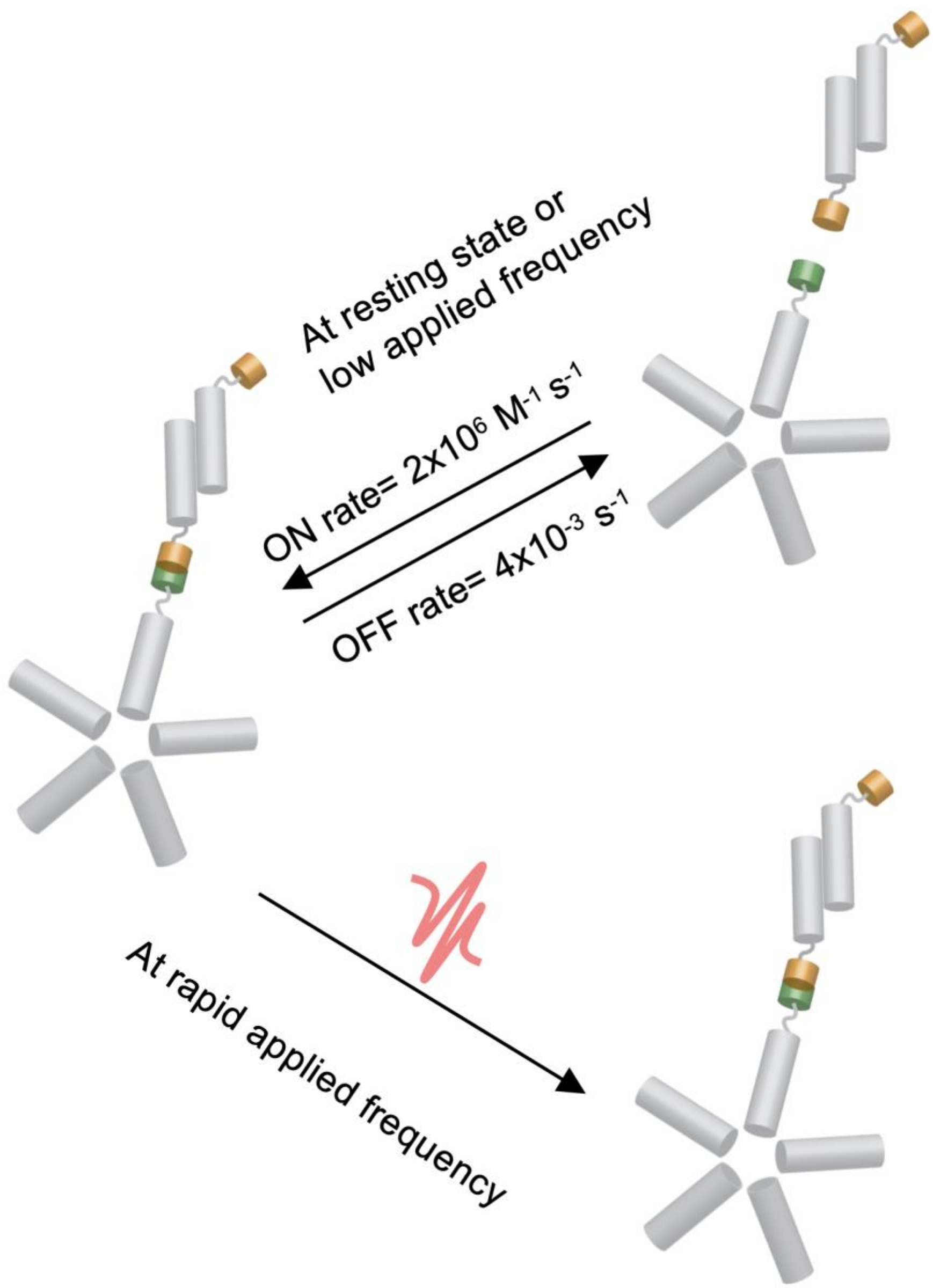

Figure S5. Model showing the noncovalent assembly between C2-5-HA and C5-5-HB at a resting state or at a state when the applied frequency is low vs when the applied frequency is rapid. At the resting state, due to the rapid ON/OFF rate of the heterodimer units, the material behaves like a liquid, whereas, if the assembly exerts a deformation frequency faster than the heterodimer LHD101A:B (HA and HB) ON/OFF rate then the intermolecular interactions get 'locked', and therefore behaves like a gel.

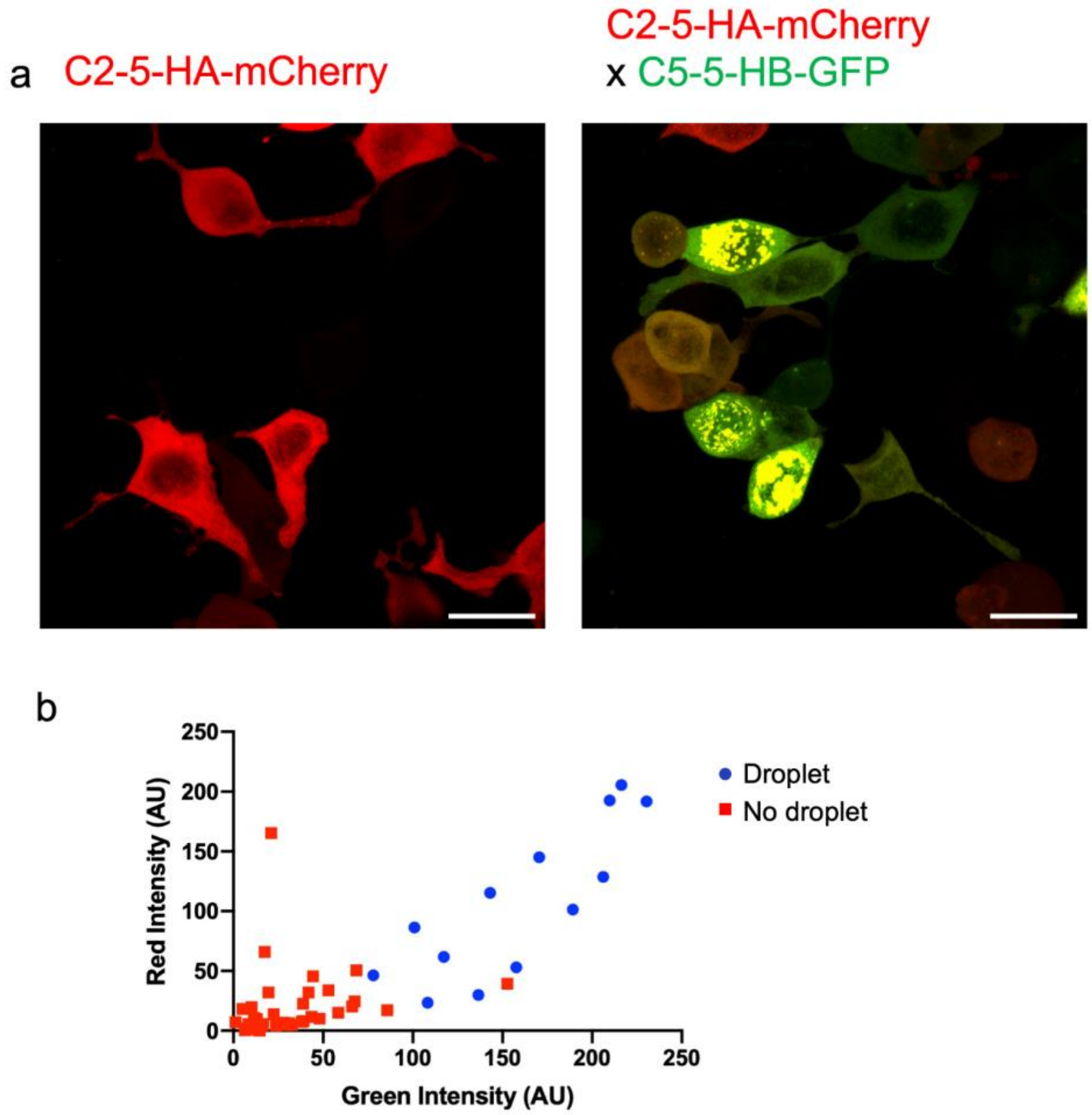

Figure S6. The phase transition from freely diffused constituents to droplet punctae. a) Expression of C2-5-HA-mCherry alone or the coexpression of C2-5-HA-mCherry and C2-5-HB-GFP. b) Droplet formation occurred only at a certain threshold expression of both the components, measured through red (mCherry) and green (GFP) fluorescent intensity.

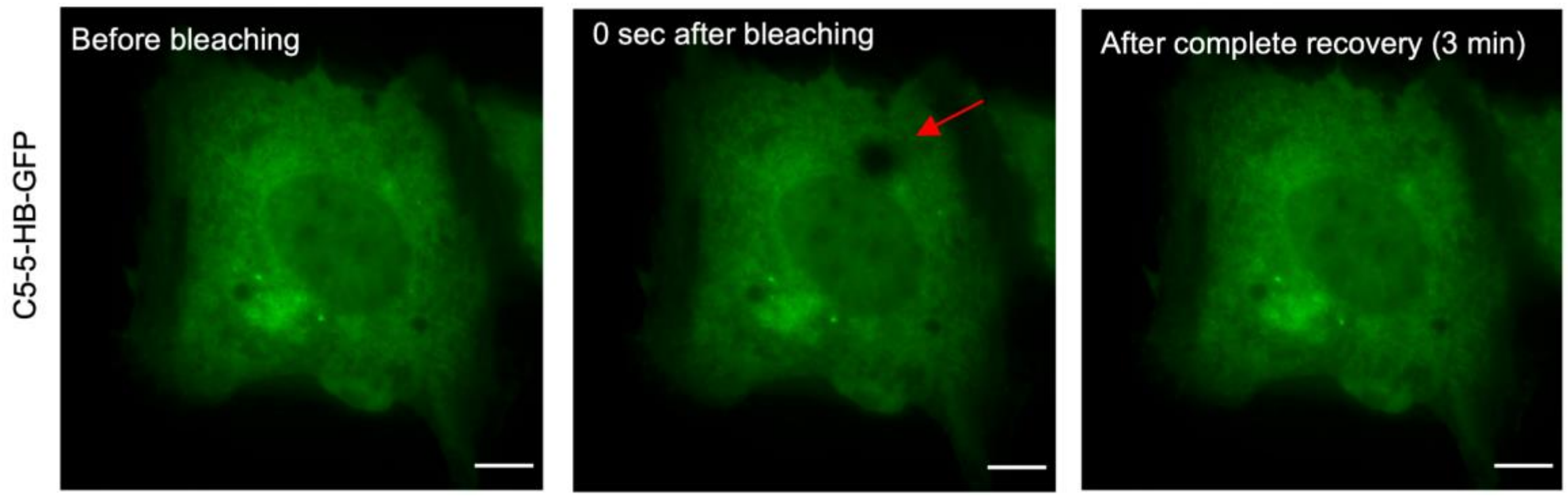

Figure S7. Fluorescence recovery after photobleaching (FRAP) experiment of the control C5-5-HB-GFP expressing cells demonstrated a rapid recovery in the absence of a droplet formation.

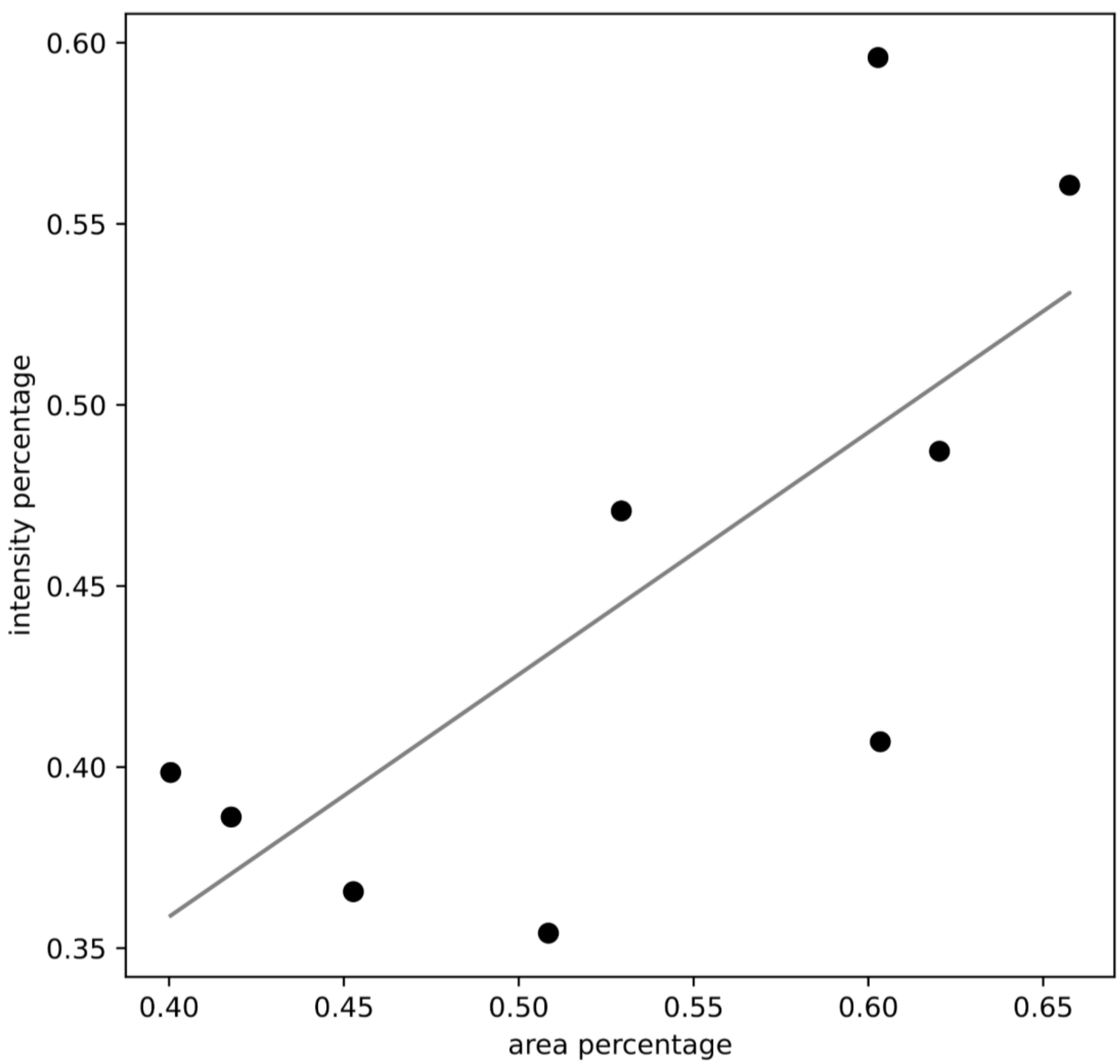

Figure S8. Measurement of bleached area versus fluorescence intensity recovery. The x-axis represents the area percentage, measured by taking the ratio of the droplet area pre and post bleach; whereas the y-axis represents the intensity percentage, measured by taking the ratio of full droplet intensity pre-bleach to the intensity post-recovery.

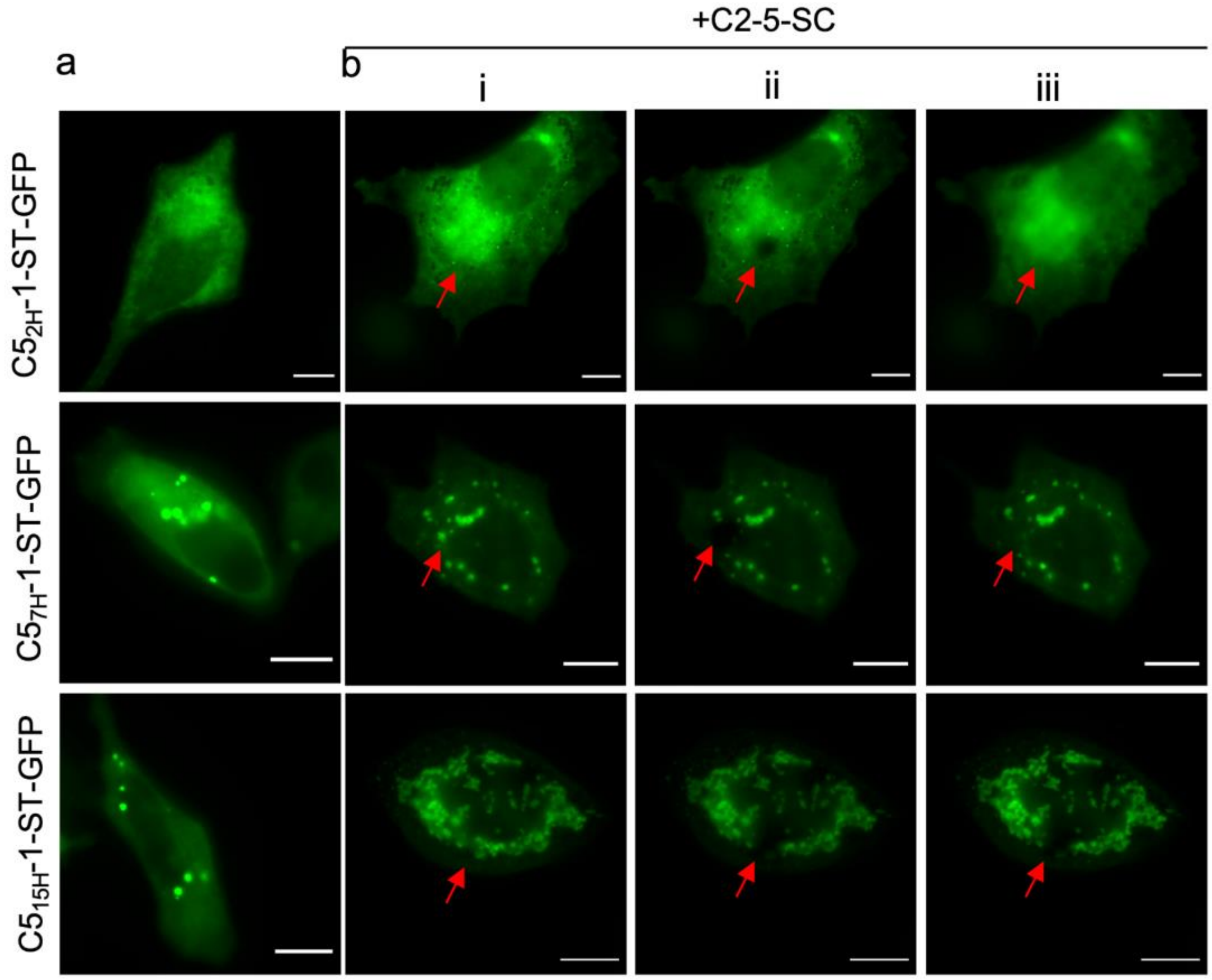

Figure S9: Fluorescence recovery after photobleaching (FRAP) experiment of the rigid arm series. a) Expression of $C5_{2H}$-1-ST-GFP, $C5_{7H}$-1-ST-GFP, and $C5_{15H}$-1-ST-GFP constructs alone, or with C2-5-SC (b). (i) Before photobleaching, (ii) right after photobleaching, and (iii) 3 minutes after photobleaching. Scale bar 10 µm.

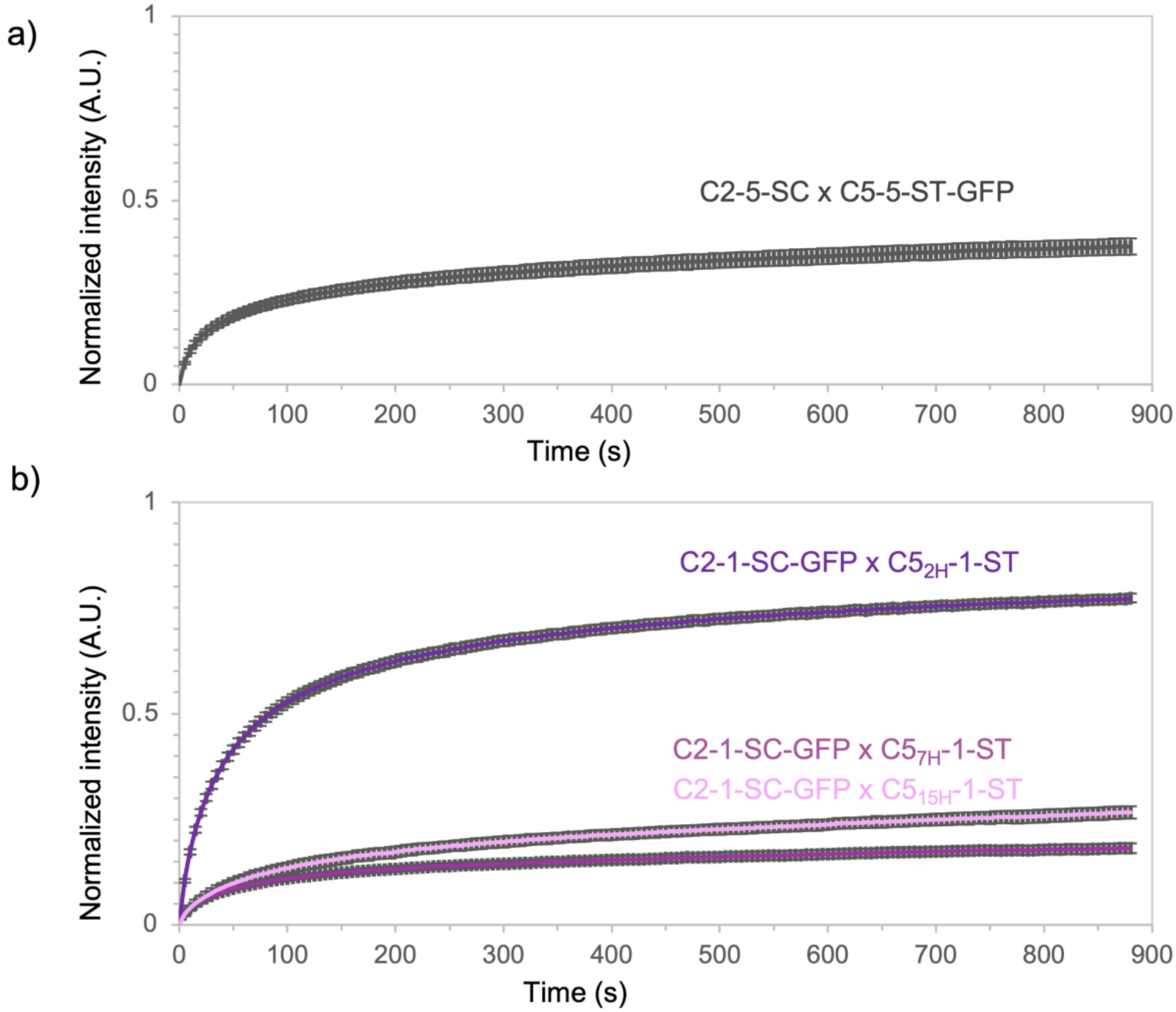

Figure S10: Fluorescence recovery after photobleaching (FRAP) experiment of bulk hydrogel formed by a flexible linker (a) and the rigid arm building block series (b). Time-dependent fluorescence recovery plot of covalent bulk hydrogel (*ex cellulo*) of (a) C2-5-SC x C5-5-ST-GFP; and (b) C2-1-SC-GFP x C5$_{2H}$-1-ST; C2-1-SC-GFP x C5$_{7H}$-1-ST; and C2-1-SC-GFP x C5$_{15H}$-1-ST.

Supplementary Tables

Table S1. Coordinates (XYZ) of components comprising C5-5-ST and C2-5-SC models. The radius of each bead of the C5 body is 1.2σ, and that of the C2 body is 0.67σ. The radius of all other beads is 0.5σ. The length of the GGS linker (*i.e.* the number of beads of the GGS linker) can be changed depending on the system.

C5-5				C2-5			
Type	x	y	z	Type	x	y	z
C5 body	0.000	-0.002	0.000	C2 anchor	-5.466	0.000	-0.059
C5 body	0.000	2.390	0.000	C2 anchor	3.934	0.000	-0.059
C5 body	0.000	4.782	0.000	C2 body	-4.795	0.000	-0.059
C5 body	2.275	0.738	0.000	C2 body	-3.452	0.000	-0.059
C5 body	4.550	1.477	0.000	C2 body	-2.109	0.000	-0.059
C5 body	1.406	-1.937	0.000	C2 body	-0.766	0.000	-0.059
C5 body	2.812	-3.872	0.000	C2 body	0.576	0.000	-0.059
C5 body	-1.406	-1.937	0.000	C2 body	1.919	0.000	-0.059
C5 body	-2.812	-3.872	0.000	C2 body	3.262	0.000	-0.059
C5 body	-2.275	0.738	0.000	GGS linker	-6.466	0.000	-0.059
C5 body	-4.550	1.477	0.000	GGS linker	-7.466	0.000	-0.059
C5 anchor	0.000	5.978	0.000	GGS linker	-8.466	0.000	-0.059
C5 anchor	5.687	1.846	0.000	GGS linker	-9.466	0.000	-0.059
C5 anchor	3.515	-4.840	0.000	GGS linker	-10.466	0.000	-0.059
C5 anchor	-3.515	-4.840	0.000	GGS linker	4.934	0.000	-0.059
C5 anchor	-5.687	1.846	0.000	GGS linker	5.934	0.000	-0.059

GGS linker	0.000	6.978	0.000	GGS linker	6.934	0.000	-0.059
GGS linker	0.000	7.978	0.000	GGS linker	7.934	0.000	-0.059
GGS linker	0.000	8.978	0.000	GGS linker	8.934	0.000	-0.059
GGS linker	0.000	9.978	0.000	SpyCatcher patch	-11.466	0.000	-0.059
GGS linker	0.000	10.978	0.000	SpyCatcher patch	-12.466	0.000	-0.059
GGS linker	6.638	2.155	0.000	SpyCatcher patch	-13.466	0.000	-0.059
GGS linker	7.589	2.464	0.000	SpyCatcher patch	-14.466	0.000	-0.059
GGS linker	8.540	2.773	0.000	SpyCatcher patch	12.934	0.000	-0.059
GGS linker	9.492	3.082	0.000	SpyCatcher patch	11.934	0.000	-0.059
GGS linker	10.443	3.391	0.000	SpyCatcher patch	10.934	0.000	-0.059
GGS linker	4.103	-5.649	0.000	SpyCatcher patch	9.934	0.000	-0.059
GGS linker	4.691	-6.458	0.000	SpyCatcher body	-11.466	0.000	1.041
GGS linker	5.278	-7.267	0.000	SpyCatcher body	-11.466	0.953	0.491
GGS linker	5.866	-8.076	0.000	SpyCatcher body	-11.466	0.953	-0.609
GGS linker	6.454	-8.885	0.000	SpyCatcher body	-11.466	-0.953	-0.609

GGS linker	-4.103	-5.649	0.000	SpyCatcher body	-11.466	-0.953	0.491
GGS linker	-4.691	-6.458	0.000	SpyCatcher body	-12.466	0.000	1.041
GGS linker	-5.278	-7.267	0.000	SpyCatcher body	-12.466	0.953	0.491
GGS linker	-5.866	-8.076	0.000	SpyCatcher body	-12.466	0.953	-0.609
GGS linker	-6.454	-8.885	0.000	SpyCatcher body	-12.466	-0.953	-0.609
GGS linker	-6.638	2.155	0.000	SpyCatcher body	-12.466	-0.953	0.491
GGS linker	-7.589	2.464	0.000	SpyCatcher body	-13.466	0.000	1.041
GGS linker	-8.540	2.773	0.000	SpyCatcher body	-13.466	0.953	0.491
GGS linker	-9.492	3.082	0.000	SpyCatcher body	-13.466	0.953	-0.609
GGS linker	-10.443	3.391	0.000	SpyCatcher body	-13.466	-0.953	-0.609
SpyTag	0.000	11.978	0.000	SpyCatcher body	-13.466	-0.953	0.491
SpyTag	0.000	12.978	0.000	SpyCatcher body	-14.466	0.000	1.041
SpyTag	0.000	13.978	0.000	SpyCatcher body	-14.466	0.953	0.491
SpyTag	0.000	14.978	0.000	SpyCatcher body	-14.466	0.953	-0.609
SpyTag	11.394	3.700	0.000	SpyCatcher body	-14.466	-0.953	-0.609
SpyTag	12.345	4.009	0.000	SpyCatcher body	-14.466	-0.953	0.491
SpyTag	13.296	4.318	0.000	SpyCatcher body	12.934	0.000	1.041

SpyTag	14.247	4.627	0.000	SpyCatcher body	12.934	0.953	0.491
SpyTag	7.042	-9.694	0.000	SpyCatcher body	12.934	0.953	-0.609
SpyTag	7.629	-10.503	0.000	SpyCatcher body	12.934	0.000	-1.159
SpyTag	8.217	-11.312	0.000	SpyCatcher body	12.934	-0.953	-0.609
SpyTag	8.805	-12.121	0.000	SpyCatcher body	12.934	-0.953	0.491
SpyTag	-7.042	-9.694	0.000	SpyCatcher body	11.934	0.000	1.041
SpyTag	-7.629	-10.503	0.000	SpyCatcher body	11.934	0.953	0.491
SpyTag	-8.217	-11.312	0.000	SpyCatcher body	11.934	0.953	-0.609
SpyTag	-8.805	-12.121	0.000	SpyCatcher body	11.934	0.000	-1.159
SpyTag	-11.394	3.700	0.000	SpyCatcher body	11.934	-0.953	-0.609
SpyTag	-12.345	4.009	0.000	SpyCatcher body	11.934	-0.953	0.491
SpyTag	-13.296	4.318	0.000	SpyCatcher body	10.934	0.000	1.041
SpyTag	-14.247	4.627	0.000	SpyCatcher body	10.934	0.953	0.491
				SpyCatcher body	10.934	0.953	-0.609
				SpyCatcher body	10.934	0.000	-1.159
				SpyCatcher body	10.934	-0.953	-0.609
				SpyCatcher body	10.934	-0.953	0.491
				SpyCatcher body	9.934	0.000	1.041
				SpyCatcher body	9.934	0.953	0.491
				SpyCatcher body	9.934	0.953	-0.609
				SpyCatcher body	9.934	0.000	-1.159

				SpyCatcher body	9.934	-0.953	-0.609
				SpyCatcher body	9.934	-0.953	0.491

Supplementary Protein Sequences

Amino acid sequence: Yellow: linker; Red: SpyCatcher; Blue: SpyTag; Orange: LHD101A (HA); Purple: LHD101B (HB); Green: Fluorescent protein

Covalent:

>C2-GGS-SpyCatcher (C2-1-SC)

MGTREEIIRELARSLAEQAELTARLERLLRELERLQREGSSDEDVRELLREIKELVREILKLIAEQILLI
AELLLAAIRSEAAELALRAIREAIELCKRSTDEELCQLLLRLALLLMELALLYPDSEAAKLALKAALEAI
ELCKQSTDEELCEELVKLAQKLIELAKRYPDSEAAKLALKAALEAIELCKQSTDEELCEELVKLAQKLIE
LAKRYPDSEEAKRALKEAKELIEQCKESTDEDECRELVKRAEELIREAKEGGSDSATHIKFSKRDIDGKE
LAGATMELRDSSGKTISTWISDGQVKDFYLMPGKYTFVETAAPDGYEIATAITFTVNEQGQVTVNGKATK
GGSWGLEHHHHHH

>C2-(GGS)$_5$-SpyCatcher (C2-5-SC)

MGTREEIIRELARSLAEQAELTARLERLLRELERLQREGSSDEDVRELLREIKELVREILKLIAEQILLI
AELLLAAIRSEAAELALRAIREAIELCKRSTDEELCQLLLRLALLLMELALLYPDSEAAKLALKAALEAI
ELCKQSTDEELCEELVKLAQKLIELAKRYPDSEAAKLALKAALEAIELCKQSTDEELCEELVKLAQKLIE
LAKRYPDSEEAKRALKEAKELIEQCKESTDEDECRELVKRAEELIREAKEGGSGGSGGSGGSGGSDSATH
IKFSKRDIDGKELAGATMELRDSSGKTISTWISDGQVKDFYLMPGKYTFVETAAPDGYEIATAITFTVNE
QGQVTVNGKATKGGSWGLEHHHHHH

>C5-(GGS)$_1$-SpyTag (C5-1-ST)

MGHHHHHHGWSGAHIVMVDAYKPTKGGSNDEKEKLKELLKRAEELAKSPDPEDLKEAVRLAEEVVRERPG
SNLAKKALEIILRAAEELAKLPDPEALKEAVKAAEKVVREQPGSNLAKKAQEIILRAAEELAKLEDEEAL
KEAIKAAEKVIELEPGSELAKEAKRIIEKAAKMLADILRKEMEKIREETEEVKKEIEESKKRPQSESAKN
LILIMQLLINQIRLLALQIRMLVLQLIL

>C5-(GGS)$_5$-SpyTag (C5-5-ST)

MGHHHHHHGWSGAHIVMVDAYKPTKGGSGGSGGSGGSGGSNDEKEKLKELLKRAEELAKSPDPEDLKEAV
RLAEEVVRERPGSNLAKKALEIILRAAEELAKLPDPEALKEAVKAAEKVVREQPGSNLAKKAQEIILRAA
EELAKLEDEEALKEAIKAAEKVIELEPGSELAKEAKRIIEKAAKMLADILRKEMEKIREETEEVKKEIEE
SKKRPQSESAKNLILIMQLLINQIRLLALQIRMLVLQLIL

>C5-(GGS)$_{10}$-SpyTag (C5-10-ST)

MGHHHHHHGWSGAHIVMVDAYKPTKGGSGGSGGSGGSGGSGGSGGSGGSGGSGGSNDEKEKLKELLKRAE
ELAKSPDPEDLKEAVRLAEEVVRERPGSNLAKKALEIILRAAEELAKLPDPEALKEAVKAAEKVVREQPG
SNLAKKAQEIILRAAEELAKLEDEEALKEAIKAAEKVIELEPGSELAKEAKRIIEKAAKMLADILRKEME
KIREETEEVKKEIEESKKRPQSESAKNLILIMQLLINQIRLLALQIRMLVLQLIL

>T33_chainA

MGEEAELAYLLGELAYKLGEYRIAIRAYRIALKRDPNNAEAWYNLGNAYYKQGDYDEAIEYYQKALELDP
NNAEAWYNLGNAYYKQGDYDEAIEYYEKALELDPENLEALQNLLNAMDKQG

>T33-(GGS)$_1$-SpyTag_chainB (T33-1-ST_chainB)

MIEEVVAEMIDILAESSKKSIEELARAADNKTTEKAVAEAIEEIARLATAAIQLIEALAKNLASEEFMAR
AISAIAELAKKAIEAIYRLADNHTTDTFMARAIAAIANLAVTAILAIAALASNHTTEEFMARAISAIAEL
AKKAIEAIYRLADNHTTDKFMAAAIEAIALLATLAILAIALLASNHTTEKFMARAIMAIAILAAKAIEAI
YRLADNHTSPTYIEKAIEAIEKIARKAIKAIEMLAKNITTEEYKEKAKKIIDIIRKLAKMAIKKLEDNRT
GGSAHIVMVDAYKPTKGSWGLEHHHHHH

>T33-(GGS)$_5$-SpyTag_chainB (T33-5-ST_chainB)

MIEEVVAEMIDILAESSKKSIEELARAADNKTTEKAVAEAIEEIARLATAAIQLIEALAKNLASEEFMAR
AISAIAELAKKAIEAIYRLADNHTTDTFMARAIAAIANLAVTAILAIAALASNHTTEEFMARAISAIAEL
AKKAIEAIYRLADNHTTDKFMAAAIEAIALLATLAILAIALLASNHTTEKFMARAIMAIAILAAKAIEAI
YRLADNHTSPTYIEKAIEAIEKIARKAIKAIEMLAKNITTEEYKEKAKKIIDIIRKLAKMAIKKLEDNRT
GGSGGSGGSGGSGGSAHIVMVDAYKPTKGSWGLEHHHHHH

>T33-(GGS)$_{10}$-SpyTag_chainB (T33-10-ST_chainB)

MIEEVVAEMIDILAESSKKSIEELARAADNKTTEKAVAEAIEEIARLATAAIQLIEALAKNLASEEFMAR
AISAIAELAKKAIEAIYRLADNHTTDTFMARAIAAIANLAVTAILAIAALASNHTTEEFMARAISAIAEL
AKKAIEAIYRLADNHTTDKFMAAAIEAIALLATLAILAIALLASNHTTEKFMARAIMAIAILAAKAIEAI
YRLADNHTSPTYIEKAIEAIEKIARKAIKAIEMLAKNITTEEYKEKAKKIIDIIRKLAKMAIKKLEDNRT
GGSGGSGGSGGSGGSGGSGGSGGSGGSGGSAHIVMVDAYKPTKGSWGLEHHHHHH

>I53_chainA

MGKYDGSKLRIGILHARWNAEIILALVLGALKRLQEFGVKRENIIIETVPGSFELPYGSKLFVEKQKRLG
KPLDAIIPIGVLIKGSTMHFEYICDSTTHQLMKLNFELGIPVIFGVLTCLTDEQAEARAGLIEGKMHNHG
EDWGAAAVEMATKFN

>I53-(GGS)$_1$-SpyTag_chainB (I53-1-ST_chainB)

MEEAELAYLLGELAYKLGEYRIAIRAYRIALKRDPNNAEAWYNLGNAYYKQGRYREAIEYYQKALELDPN
NAEAWYNLGNAYYERGEYEEAIEYYRKALRLDPNNADAMQNLLNAKMREEGGSAHIVMVDAYKPTKGSWG
LEHHHHHH

>I53-(GGS)$_5$-SpyTag_chainB (I53-5-ST_chainB)

MEEAELAYLLGELAYKLGEYRIAIRAYRIALKRDPNNAEAWYNLGNAYYKQGRYREAIEYYQKALELDPN
NAEAWYNLGNAYYERGEYEEAIEYYRKALRLDPNNADAMQNLLNAKMREEGGSGGSGGSGGSGGSAHIVM
VDAYKPTKGSWGLEHHHHHH

>I53-(GGS)$_{10}$-SpyTag_chainB (I53-10-ST_chainB)

MEEAELAYLLGELAYKLGEYRIAIRAYRIALKRDPNNAEAWYNLGNAYYKQGRYREAIEYYQKALELDPN
NAEAWYNLGNAYYERGEYEEAIEYYRKALRLDPNNADAMQNLLNAKMREEGGSGGSGGSGGSGGSGGSGG
SGGSGGSGGSAHIVMVDAYKPTKGSWGLEHHHHHH

>C5$_{2H}$-(GGS)$_1$-SpyTag (C5$_{2H}$-1-ST)

MGHHHHHHGWSGAHIVMVDAYKPTKGGSTRRKQEMKRLKKEMEKIREETEEVKKEIEESKKRPQSESAKN
LILIMQLLINQIRLLALQIRMLALQLQE

>C5$_{7H}$-(GGS)$_1$-SpyTag (C5$_{7H}$-1-ST)

MGHHHHHHGWSGAHIVMVDAYKPTK**GGS**SDLQEVADRIVEQLKREGRSPEEARKEARRLIEEIKQSAGGD
SELIEVAVRIVKFLEEAGMSPSEAAKVAVELIERIRRAAGGDSELIEKAVRIVRRLERRGLSPAEAAKIA
VAIIAAEVLSREAEKIREETEEVKKEIEESKKRPQSESAKNLILIMQLLINQIRLLALQIQMLRLQLEL

>C5$_{15H}$-(GGS)$_1$-SpyTag (C5$_{15H}$-1-ST)

MGHHHHHHGWSGAHIVMVDAYKPTKGGSSDLQEVADRIVEQLKREGRSPEEARKEARRLIEEIKQSAGGD
SELIEVAVRIVKELEEQGRSPSEAAKEAVELIERIRRAAGGDSELIEVAVRIVKELEEQGRSPSEAAKEA
VELIERIRRAAGGDSELIEVAVRIVKELEEQGRSPSEAAKEAVELIERIRRAAGGDSELIEVAVRIVKEL
EEQGRSPSEAAKEAVELIERIRRAAGGDSELIEVAVRIVKFLEEAGMSPSEAAKVAVELIERIRRAAGGD
SELIEKAVRIVRRLERRGLSPAEAAKIAVAIIAAEVLSREAEKIREETEEVKKEIEESKKRPQSESAKNL
ILIMQLLINQIRLLALQIQMLRLQLEL

Noncovalent:

>C2-(GGS)$_5$-LHD101A (C2-5-HA)

MGTREEIIRELARSLAEQAELTARLERLLRELERLQREGSSDEDVRELLREIKELVREILKLIAEQILLI
AELLLAAIRSEAAELALRAIREAIELCKRSTDEELCQLLLRLALLLMELALLYPDSEAAKLALKAALEAI
ELCKQSTDEELCEELVKLAQKLIELAKRYPDSEAAKLALKAALEAIELCKQSTDEELCEELVKLAQKLIE

LAKRYPDSEEAKRALKEAKELIEQCKESTDEDECRELVKRAEELIREAKEGGSGGSGGSGGSGGSGRQEK
VLKSIEETVRKMGVTMETHRSGNEVKVVIKGLHIKQQRQLYRDVRETSKKQGVETEIEVEGDTVTIVVRE
GSWGLEHHHHHH

>C5-(GGS)$_5$-LHD101B (C5-5-HB)

MGHHHHHHGWSGGRQEKVLKSIEETVRKMGVTMETHRSGNEVKVVIKGLHESQQEQLLEDVLRTAEKQGV
RVRIRFKGDTVTIVVREGGSGGSGGSGGSGGSNDEKEKLKELLKRAEELAKSPDPEDLKEAVRLAEEVVR
ERPGSNLAKKALEIILRAAEELAKLPDPEALKEAVKAAEKVVREQPGSNLAKKAQEIILRAAEELAKLED
EEALKEAIKAAEKVIELEPGSELAKEAKRIIEKAAKMLADILRKEMEKIREETEEVKKEIEESKKRPQSE
SAKNLILIMQLLINQIRLLALQIRMLVLQLIL

GFP/mCherry fusion proteins:

>C5-(GGS)$_5$-SpyTag-GFP (C5-5-ST-GFP)

MSKGEELFTGVVPILVELDGDVNGHKFSVSGEGEGDATYGKLTLKFICTTGKLPVPWPTLVTTLTYGVQC
FSRYPDHMKQHDFFKSAMPEGYVQERTIFFKDDGNYKTRAEVKFEGDTLVNRIELKGIDFKEDGNILGHK
LEYNYNSHNVYIMADKQKNGIKVNFKIRHNIEDGSVQLADHYQQNTPIGDGPVLLPDNHYLSTQSALSKD
PNEKRDHMVLLEFVTAAGITLGMDELYKGGSGGSGGSGGSGGSAHIVMVDAYKPTKGGSGGSGGSGGSGG
SNDEKEKLKELLKRAEELAKSPDPEDLKEAVRLAEEVVRERPGSNLAKKALEIILRAAEELAKLPDPEAL
KEAVKAAEKVVREQPGSNLAKKAQEIILRAAEELAKLEDEEALKEAIKAAEKVIELEPGSELAKEAKRII
EKAAKMLADILRKEMEKIREETEEVKKEIEESKKRPQSESAKNLILIMQLLINQIRLLALQIRMLVLQLI
L

>C5-(GGS)$_5$-LHD101B-GFP (C5-5-HB-GFP)

MSKGEELFTGVVPILVELDGDVNGHKFSVSGEGEGDATYGKLTLKFICTTGKLPVPWPTLVTTLTYGVQC
FSRYPDHMKQHDFFKSAMPEGYVQERTIFFKDDGNYKTRAEVKFEGDTLVNRIELKGIDFKEDGNILGHK
LEYNYNSHNVYIMADKQKNGIKVNFKIRHNIEDGSVQLADHYQQNTPIGDGPVLLPDNHYLSTQSALSKD
PNEKRDHMVLLEFVTAAGITLGMDELYKGGSGGSGGSGGSGGSGRQEKVLKSIEETVRKMGVTMETHRSG
NEVKVVIKGLHESQQEQLLEDVLRTAEKQGVRVRIRFKGDTVTIVVREGGSGGSGGSGGSGGSNDEKEKL
KELLKRAEELAKSPDPEDLKEAVRLAEEVVRERPGSNLAKKALEIILRAAEELAKLPDPEALKEAVKAAE
KVVREQPGSNLAKKAQEIILRAAEELAKLEDEEALKEAIKAAEKVIELEPGSELAKEAKRIIEKAAKMLA
DILRKEMEKIREETEEVKKEIEESKKRPQSESAKNLILIMQLLINQIRLLALQIRMLVLQLIL

>C2-GGS-SpyCatcher-mCherry (C2-1-SC-mCherry)

MGTREEIIRELARSLAEQAELTARLERLLRELERLQREGSSDEDVRELLREIKELVREILKLIAEQILLI
AELLLAAIRSEAAELALRAIREAIELCKRSTDEELCQLLLRLALLLMELALLYPDSEAAKLALKAALEAI
ELCKQSTDEELCEELVKLAQKLIELAKRYPDSEAAKLALKAALEAIELCKQSTDEELCEELVKLAQKLIE
LAKRYPDSEEAKRALKEAKELIEQCKESTDEDECRELVKRAEELIREAKEGGSDSATHIKFSKRDIDGKE
LAGATMELRDSSGKTISTWISDGQVKDFYLMPGKYTFVETAAPDGYEIATAITFTVNEQGQVTVNGKATK

GGGSGGSGGSGGSGGSVSKGEEDNMAIIKEFMRFKVHMEGSVNGHEFEIEGEGEGRPYEGTQTAKLKVTK
GGPLPFAWDILSPQFMYGSKAYVKHPADIPDYLKLSFPEGFKWERVMNFEDGGVVTVTQDSSLQDGEFIY
KVKLRGTNFPSDGPVMQKKTMGWEASSERMYPEDGALKGEIKQRLKLKDGGHYDAEVKTTYKAKKPVQLP
GAYNVNIKLDITSHNEDYTIVEQYERAEGRHSTGGMDELYK

>$C5_{2H}$-GGS-SpyTag-GFP ($C5_{2H}$-1-ST-GFP)

MSKGEELFTGVVPILVELDGDVNGHKFSVSGEGEGDATYGKLTLKFICTTGKLPVPWPTLVTTLTYGVQC
FSRYPDHMKQHDFFKSAMPEGYVQERTIFFKDDGNYKTRAEVKFEGDTLVNRIELKGIDFKEDGNILGHK
LEYNYNSHNVYIMADKQKNGIKVNFKIRHNIEDGSVQLADHYQQNTPIGDGPVLLPDNHYLSTQSALSKD
PNEKRDHMVLLEFVTAAGITLGMDELYKGGSGGSGGSGGSGGSAHIVMVDAYKPTKGGSTRRKQEMKRLK
KEMEKIREETEEVKKEIEESKKRPQSESAKNLILIMQLLINQIRLLALQIRMLALQLQE

>$C5_{7H}$-GGS-SpyTag-GFP ($C5_{7H}$-1-ST-GFP)

MSKGEELFTGVVPILVELDGDVNGHKFSVSGEGEGDATYGKLTLKFICTTGKLPVPWPTLVTTLTYGVQC
FSRYPDHMKQHDFFKSAMPEGYVQERTIFFKDDGNYKTRAEVKFEGDTLVNRIELKGIDFKEDGNILGHK
LEYNYNSHNVYIMADKQKNGIKVNFKIRHNIEDGSVQLADHYQQNTPIGDGPVLLPDNHYLSTQSALSKD
PNEKRDHMVLLEFVTAAGITLGMDELYKGGSGGSGGSGGSGGSAHIVMVDAYKPTKGGSSDLQEVADRIV
EQLKREGRSPEEARKEARRLIEEIKQSAGGDSELIEVAVRIVKFLEEAGMSPSEAAKVAVELIERIRRAA
GGDSELIEKAVRIVRRLERRGLSPAEAAKIAVAIIAAEVLSREAEKIREETEEVKKEIEESKKRPQSESA
KNLILIMQLLINQIRLLALQIQMLRLQLEL

>$C5_{15H}$-GGS-SpyTag-GFP ($C5_{15H}$-1-ST-GFP)

MSKGEELFTGVVPILVELDGDVNGHKFSVSGEGEGDATYGKLTLKFICTTGKLPVPWPTLVTTLTYGVQC
FSRYPDHMKQHDFFKSAMPEGYVQERTIFFKDDGNYKTRAEVKFEGDTLVNRIELKGIDFKEDGNILGHK
LEYNYNSHNVYIMADKQKNGIKVNFKIRHNIEDGSVQLADHYQQNTPIGDGPVLLPDNHYLSTQSALSKD
PNEKRDHMVLLEFVTAAGITLGMDELYKGGSGGSGGSGGSGGSAHIVMVDAYKPTKGGSSDLQEVADRIV
EQLKREGRSPEEARKEARRLIEEIKQSAGGDSELIEVAVRIVKELEEQGRSPSEAAKEAVELIERIRRAA
GGDSELIEVAVRIVKELEEQGRSPSEAAKEAVELIERIRRAAGGDSELIEVAVRIVKELEEQGRSPSEAA
KEAVELIERIRRAAGGDSELIEVAVRIVKELEEQGRSPSEAAKEAVELIERIRRAAGGDSELIEVAVRIV
KFLEEAGMSPSEAAKVAVELIERIRRAAGGDSELIEKAVRIVRRLERRGLSPAEAAKIAVAIIAAEVLSR
EAEKIREETEEVKKEIEESKKRPQSESAKNLILIMQLLINQIRLLALQIQMLRLQLEL

>C2-$(GGS)_5$-LHD101A-mCherry (C2-5-HA-mCherry)

MGTREEIIRELARSLAEQAELTARLERLLRELERLQREGSSDEDVRELLREIKELVREILKLIAEQILLI
AELLLAAIRSEAAELALRAIREAIELCKRSTDEELCQLLLRLALLLMELALLYPDSEAAKLALKAALEAI
ELCKQSTDEELCEELVKLAQKLIELAKRYPDSEAAKLALKAALEAIELCKQSTDEELCEELVKLAQKLIE
LAKRYPDSEEAKRALKEAKELIEQCKESTDEDECRELVKRAEELIREAKEGGSGGSGGSGGSGGSGRQEK
VLKSIEETVRKMGVTMETHRSGNEVKVVIKGLHIKQQRQLYRDVRETSKKQGVETEIEVEGDTVTIVVRE
GGSGGSGGSGGSGGSVSKGEEDNMAIIKEFMRFKVHMEGSVNGHEFEIEGEGEGRPYEGTQTAKLKVTKG
GPLPFAWDILSPQFMYGSKAYVKHPADIPDYLKLSFPEGFKWERVMNFEDGGVVTVTQDSSLQDGEFIYK
VKLRGTNFPSDGPVMQKKTMGWEASSERMYPEDGALKGEIKQRLKLKDGGHYDAEVKTTYKAKKPVQLPG
AYNVNIKLDITSHNEDYTIVEQYERAEGRHSTGGMDELYK

>C5-$(GGS)_5$-LHD101B-Lifeact-GFP (C5-5-HB-Lifeact-GFP) [Lifeact in cyan]

MGVADLIKKFESISKEEGDPPVATMVSKGEELFTGVVPILVELDGDVNGHKFSVSGEGEGDATYGKLTLK
FICTTGKLPVPWPTLVTTLTYGVQCFSRYPDHMKQHDFFKSAMPEGYVQERTIFFKDDGNYKTRAEVKFE
GDTLVNRIELKGIDFKEDGNILGHKLEYNYNSHNVYIMADKQKNGIKVNFKIRHNIEDGSVQLADHYQQN
TPIGDGPVLLPDNHYLSTQSALSKDPNEKRDHMVLKEFVTAAGITLGMDELYKGGSGGSGGSGGSGGSGR
QEKVLKSIEETVRKMGVTMETHRSGNEVKVVIKGLHESQQEQLLEDVLRTAEKQGVRVRIRFKGDTVTIV
VREGGSGGSGGSGGSGGSNDEKEKLKELLKRAEELAKSPDPEDLKEAVRLAEEVVRERPGSNLAKKALEI
ILRAAEELAKLPDPEALKEAVKAAEKVVREQPGSNLAKKAQEIILRAAEELAKLEDEEALKEAIKAAEKV
IELEPGSELAKEAKRIIEKAAKMLADILRKEMEKIREETEEVKKEIEESKKRPQSESAKNLILIMQLLIN
QIRLLALQIRMLVLQLIL

>C2-GGS-SpyCatcher-GFP (C2-1-SC-GFP) (bacterial expression)

MGTREEIIRELARSLAEQAELTARLERLLRELERLQREGSSDEDVRELLREIKELVREILKLIAEQILLI
AELLLAAIRSEAAELALRAIREAIELCKRSTDEELCQLLLRLALLLMELALLYPDSEAAKLALKAALEAI
ELCKQSTDEELCEELVKLAQKLIELAKRYPDSEAAKLALKAALEAIELCKQSTDEELCEELVKLAQKLIE
LAKRYPDSEEAKRALKEAKELIEQCKESTDEDECRELVKRAEELIREAKEGGSDSATHIKFSKRDIDGKE
LAGATMELRDSSGKTISTWISDGQVKDFYLMPGKYTFVETAAPDGYEIATAITFTVNEQGQVTVNGKATK
GGGSGGSGGSGGSGGSMSKGEELFTGVVPILVELDGDVNGHKFSVRGEGEGDATNGKLTLKFICTTGKLP
VPWPTLVTTLTYGVQCFARYPDHMKQHDFFKSAMPEGYVQERTISFKDDGTYKTRAEVKFEGDTLVNRIE
LKGIDFKEDGNILGHKLEYNFNSHNVYITADKQKNGIKANFKIRHNVEDGSVQLADHYQQNTPIGDGPVL
LPDNHYLSTQSVLSKDPNEKRDHMVLLEFVTAAGITHGMDELYKLEHHHHHH

>C5-$(GGS)_5$-SpyTag-GFP (C5-5-ST-GFP) (bacterial expression)

MSKGEELFTGVVPILVELDGDVNGHKFSVRGEGEGDATNGKLTLKFICTTGKLPVPWPTLVTTLTYGVQC
FARYPDHMKQHDFFKSAMPEGYVQERTISFKDDGTYKTRAEVKFEGDTLVNRIELKGIDFKEDGNILGHK
LEYNFNSHNVYITADKQKNGIKANFKIRHNVEDGSVQLADHYQQNTPIGDGPVLLPDNHYLSTQSVLSKD
PNEKRDHMVLLEFVTAAGITHGMDELYKGGSGGSGGSGGSGGSAHIVMVDAYKPTKGGSGGSGGSGGSGG
SNDEKEKLKELLKRAEELAKSPDPEDLKEAVRLAEEVVRERPGSNLAKKALEIILRAAEELAKLPDPEAL
KEAVKAAEKVVREQPGSNLAKKAQEIILRAAEELAKLEDEEALKEAIKAAEKVIELEPGSELAKEAKRII
EKAAKMLADILRKEMEKIREETEEVKKEIEESKKRPQSESAKNLILIMQLLINQIRLLALQIRMLVLQLI
LLEHHHHHH
